# pH-resilient Cu/Si_3_N_4_ catalyst for efficient heterogeneous Fenton mineralization of methylene blue

**DOI:** 10.1039/d6ra06509k

**Published:** 2026-10-23

**Authors:** Sajid Hussain, Eleonora Aneggi, Walter Baratta, Daniele Zuccaccia, Jordi Llorca, Alessandro Trovarelli, Michele Modesti, Daniele Goi

**Affiliations:** a Dipartimento Politecnico di Ingegneria e Architettura, Università di Udine, INSTM Udine Via del Cotonificio 108 33100 Udine Italy; b Dipartimento di Scienze AgroAlimentari, Ambientali e Animali, Sezione di Chimica, Università di Udine, INSTM Udine Via del Cotonificio 108 33100 Udine Italy eleonora.aneggi@uniud.it; c Department of Chemical Engineering, Barcelona Research Center in Multiscale Science and Engineering, Universitat Politècnica de Catalunya, EEBE Eduard Maristany 10-14 08019 Barcelona Spain; d Dipartimento di Ingegneria Industriale, Università di Padova Via Gradenigo, 6/a 35131 Padova Italy

## Abstract

Heterogeneous Fenton oxidation is an effective strategy for the removal of organic contaminants from water; however, its practical application is often hindered by narrow pH operating windows and catalyst instability. In this work, metal-supported silicon nitride catalysts (M/Si_3_N_4_, M = Cu, Fe, Co, Zn) were investigated for the mineralization of Methylene Blue (MB) over a wide pH range (3–8). A novel descriptor, the Heterogeneous Fenton Potential (HFP), is proposed as a simple tool for the preliminary screening of catalytic systems, enabling a qualitative evaluation of their ability to generate reactive oxidizing species from H_2_O_2_. Among the investigated catalysts, Cu/Si_3_N_4_ showed superior and pH-resilient performance, achieving nearly complete MB degradation within 5 min and up to 75% Total Organic Carbon (TOC) removal at near-neutral pH (5–6). In contrast, Fe-, Co-, and Zn-based systems exhibited a significant decrease in activity at pH ≥ 6. The enhanced performance of Cu/Si_3_N_4_ is attributed to the high dispersion of Cu species on the Si_3_N_4_ support, which facilitates efficient H_2_O_2_ activation and rapid generation of reactive radicals. These results highlight Cu/Si_3_N_4_ as a promising catalyst for water treatment applications, combining wide pH operability, high mineralization efficiency, and potential reusability. The proposed approach provides a promising pathway to mitigate the intrinsic pH limitations of conventional Fenton processes, supporting their further development for wastewater treatment.

## Abstract 2

Cu/Si_3_N_4_ enables pH-resilient heterogeneous Fenton oxidation by efficiently activating H_2_O_2_, achieving 75% TOC removal during methylene blue mineralization at pH 6.

## Graphical abstract

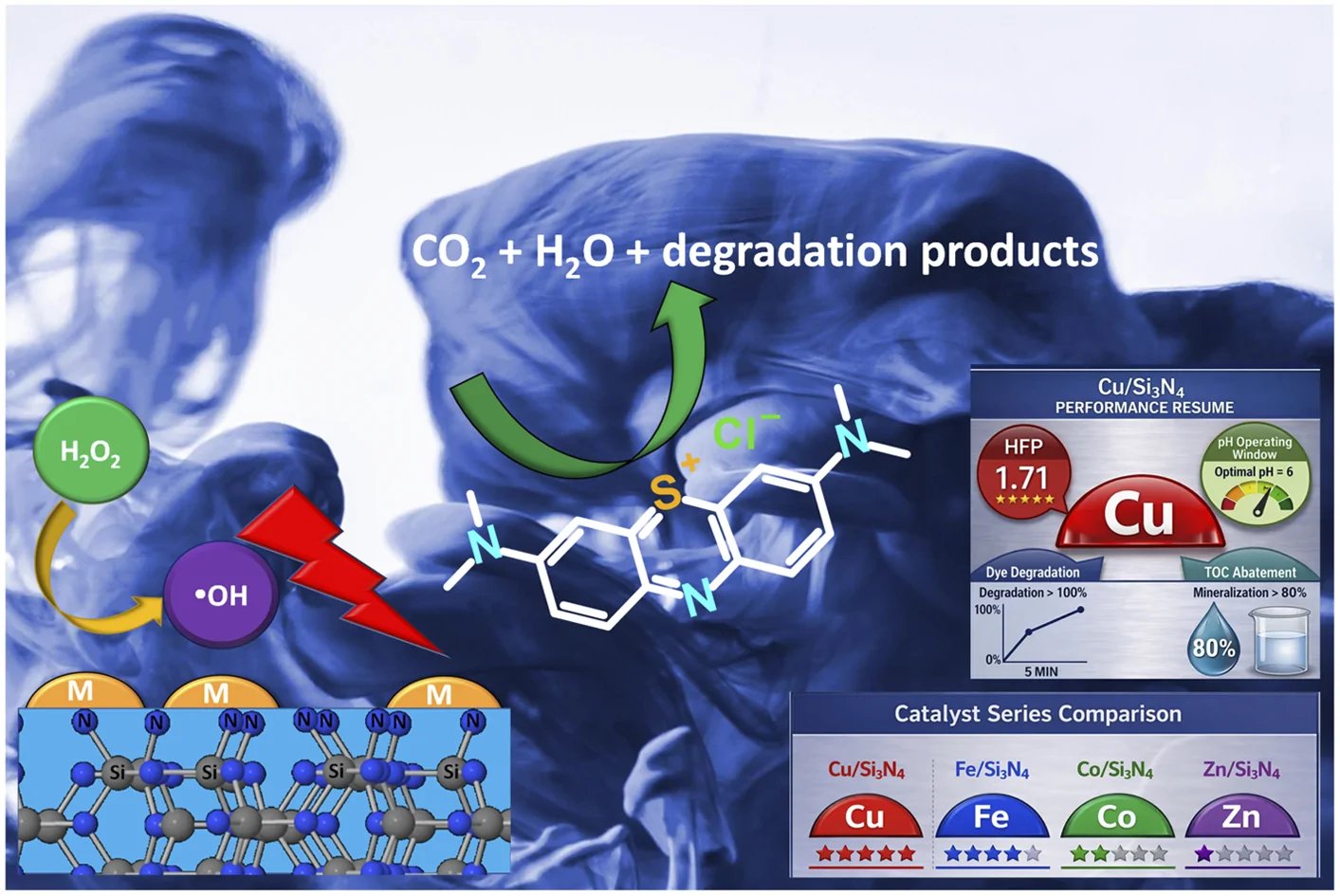

## Introduction

Water pollution originating from intensified industrial and urban activities remains a critical challenge for sustainable water resource management.^1^ Among diverse organic contaminants, synthetic dyes are particularly recalcitrant due to their complex aromatic structures, high chemical stability, and resistance to conventional biological treatment.^2^ The discharge of these compounds into aquatic ecosystems impairs light penetration and inhibits photosynthetic activity, while their potential bioaccumulation poses chronic risks to both ecological stability and human health.^3–5^ Methylene blue (MB), a representative cationic dye widely employed in the textile, pharmaceutical, and printing sectors, is frequently used as a model pollutant due to its prevalence in industrial effluents.^6^ While conventional technologies, including adsorption,^7–10^ coagulation, photocatalysis,^11–13^ and biological processes,^14^ are effective for primary treatment, they often facilitate a phase transfer of pollutants rather than complete destruction, or they struggle with the chemical stability of highly stable organic molecules.^15^

Advanced oxidation processes (AOPs) have emerged as robust alternatives for the mineralization of recalcitrant pollutants.^16–21^ These technologies rely on the *in situ* generation of highly reactive oxygen species, particularly hydroxyl radicals (˙OH), which exhibit strong oxidation potential and can non-selectively degrade a wide range of organic contaminants into smaller molecules or ultimately mineralize them into CO_2_ and H_2_O.^17,22–26^ The Fenton reaction, involving the catalytic decomposition of hydrogen peroxide by ferrous ions, is one of the most widely implemented AOPs due to its high efficiency, operational simplicity, and relatively low cost. However, homogeneous Fenton systems are hindered by stringent acidic pH requirements (pH 2.5–3.5), the continuous consumption of reagents, and the generation of secondary iron-sludge, which complicates post-treatment and increases operational costs.^27,28^ To mitigate these issues, heterogeneous Fenton-like catalysts have been developed by immobilizing active metal centers onto solid supports, enabling catalyst recovery and expanding the operating pH range.^29–34^ Despite these advancements, many heterogeneous systems still face significant hurdles, including active metal leaching, mass transfer limitations, and surface deactivation over successive cycles, which limit large scale implementation.^29^ Furthermore, the design of sustainable catalysts necessitates environmentally benign and cost-effective synthesis methods.^3,15^

Transition metals such as Fe and Cu are among the most studied catalytic centers for heterogeneous Fenton processes due to their ability to undergo redox cycling and activate hydrogen peroxide to generate reactive radicals.^29,35–40^ In liquid-phase reactions, the support must exhibit high chemical stability, resistance to hydrolysis, and strong metal–support interactions to prevent the leaching of active species. The catalytic performance of these materials is intrinsically linked to the physicochemical properties of the support. While traditional metal oxides like zirconia and alumina have been extensively studied,^38,41–44^ there is a pressing need for supports that offer superior chemical robustness and electronic modulation of the active sites.

Silicon nitride (Si_3_N_4_) represents a promising, yet comparatively underexplored, support for aqueous-phase AOPs. It has been reported to exhibit good corrosion resistance in acidic aqueous environments, although its stability may depend on factors such as the composition and microstructure of the grain-boundary phase.^45,46^ In addition, its distinctive surface chemistry characterized by nitrogen-containing groups may contribute to metal–support interactions and influence electron transfer processes during the redox cycling of transition metals.^47,48^ In view of its favorable chemical, mechanical, and thermal properties, Si_3_N_4_ was selected in this work as a promising support for transition-metal active phases in aqueous catalytic environments. The number of studies specifically addressing Si_3_N_4_-based materials for the removal of organic pollutants from aqueous media remains relatively limited and related to multicomponent photocatalysts. For example, Sharma *et al.* reported a Zr_2_Ni_1_Cu_7_/Si_3_N_4_ composite for the visible-light-assisted degradation of methylene blue, where the addition of H_2_O_2_ enhanced the degradation efficiency and hydroxyl radicals were identified as the major reactive species.^49^ In this work, a series of transition-metal catalysts (Cu, Fe, Co, and Zn) supported on silicon nitride were synthesized and investigated for the heterogeneous Fenton-like degradation of methylene blue. The catalysts were prepared by an environmentally friendly incipient wetness impregnation method and systematically evaluated in terms of catalytic activity, mineralization efficiency, and stability. Despite the large number of heterogeneous Fenton catalysts reported in the literature, studies investigating the influence of nitride-based supports on the stability of transition metal active sites in aqueous oxidation systems remains relatively scarce compared with the extensive literature available for conventional oxide- and carbon-based catalysts and photocatalysts. In particular, the potential of silicon nitride as a robust support capable of operating under near-neutral pH conditions has not been systematically investigated. In this system, the removal of methylene blue does not occur through direct interaction with the Si_3_N_4_ support, but through heterogeneous Fenton-like oxidation driven by the supported metal oxides. MB mineralization proceeds *via* reactive oxygen species generated through the redox cycling of the Cu, Fe, Co, and Zn surface species, while Si_3_N_4_ contributes by providing a chemically robust support that stabilizes highly dispersed active sites and prevents metal leaching under near-neutral conditions.

A significant novelty of this research is the introduction of a new parameter, the Heterogeneous Fenton Potential (HFP), an empirical composite index that integrates hydrogen peroxide consumption with the extent of mineralization, providing a practical measure of the effective utilization of the oxidant toward TOC removal under given reaction conditions. This parameter enables a more comprehensive evaluation of heterogeneous Fenton catalysts by integrating oxidant consumption with mineralization performance, thereby offering a useful tool for catalyst screening and process optimization. The catalytic performance of the M/Si_3_N_4_ systems was investigated by analyzing the effects of key operational parameters, including pH, catalyst dosage, oxidant concentration, pollutant loading, and temperature. In particular, the Cu/Si_3_N_4_ catalyst demonstrated remarkable activity under near-neutral conditions, thereby addressing the long-standing pH limitations of traditional Fenton chemistry. These findings highlight the potential of silicon nitride-supported catalysts as a new class of materials for efficient and sustainable wastewater treatment.

## Experimental

### Materials

All catalysts were synthesized using silicon nitride (Si_3_N_4_) as the support material. Transition metal doped catalysts (5 wt%) were prepared by the incipient wetness impregnation method, employing β-phase Si_3_N_4_ (<10 µm, Sigma-Aldrich) and aqueous solutions of the corresponding metal nitrates (reagent grade, ≥98%, Sigma-Aldrich): cobalt(ii) nitrate hexahydrate (Co(NO_3_)_2_·6H_2_O), copper(ii) nitrate hemi(pentahydrate) (Cu(NO_3_)_2_·2.5H_2_O), iron(iii) nitrate nonahydrate (Fe(NO_3_)_3_·9H_2_O) and zinc nitrate hexahydrate (Zn(NO_3_)_2_·6H_2_O). The metal nitrate solutions were prepared in deionized water and added dropwise to the Si_3_N_4_ under continuous stirring to ensure homogeneous impregnation. Their concentration was calculated according to the pore volume of β-Si_3_N_4_ and the nominal metal loading (5 wt%) required for incipient wetness impregnation. The resulting mixtures were dried overnight at 100 °C and subsequently calcined at 500 °C for 3 h.

### Catalyst's characterization

Surface area of the samples was measured according to the BET method by nitrogen adsorption at −196 °C, using a Tristar 3000 gas adsorption analyzer (Micromeritics). Structural features of the catalysts were characterized by X-ray diffraction (XRD). Philips X'Pert diffractometer was used for collecting XRD profiles (40 kV and 40 mA using Ni-filtered Cu-Kα radiation) in the range 20–80° (step size of 0.02° and a counting time of 20 s per angular abscissa). Phase identification was carried out by the Philips X'Pert HighScore software. Rietveld refinement of XRD patterns was performed by means of GSAS-EXPGUI program.^50,51^ Raman spectra were collected with a Xplora Plus Micro-Raman system (Horiba, Kyoto, Japan) equipped with a cooled CCD detector (−60 °C) and edge filter. The samples were excited with the 532 nm radiation. The spectral resolution was 1 cm^−1^ and the spectra acquisition was of 12 accumulations of 10 s with a 50× LWD objective. The optical images were collected with an integrated microscope Olympus BX43 (Olympus, Tokyo, Japan) with a 10× objective. High-angle annular dark-field scanning transmission electron microscopy (HAADF-STEM) and energy-dispersive X-ray analysis (EDX) were performed using a ThermoFisher Scientific Spectra 300 monochromated electron microscope with double aberration correction, operated at 200 kV. The sample was dispersed in methanol, and a drop of the supernatant was placed on a lacey-carbon grid.

### Fenton like oxidation

Aqueous solutions of methylene blue (MB) were prepared by dissolving 10 mg L^−1^ of MB (Sigma-Aldrich) in ultra-pure water. The pH of the MB solution was adjusted to the required value by the addition of 0.2 M NaOH/H_2_SO_4_ (Sigma-Aldrich); no fixed ratio was used, as the adjustment depends solely on the target pH. The MB aqueous samples (100 mL) were loaded with 100 mg L^−1^ of the catalysts and heated at 70 °C under reflux and continuous stirring conditions (500 rpm), using an Omni multistage reaction station. The reaction mixture was prepared by adding 57 mL L^−1^ of a 3% (w/w) H_2_O_2_ stock solution, corresponding to an initial H_2_O_2_ concentration of approximately 50 mM in a final reaction volume of 100 mL (*i.e.*, 5 mL of the 3% solution per 100 mL batch). The Fenton like oxidation reaction was carried out for 30 minutes collecting samples every 5-minute intervals, and maintaining the reaction mixture at 70 °C under reflux for the entire duration of the experiment. The samples were centrifuged at 5000 rpm using Eppendorf Centrifuge 5804 R for 10 minutes followed by filtration of the supernatant solutions through cellulose acetate membrane filters (0.45 µm pore size) and collecting the liquid in plastic vials for UV-VIS and TOC analysis. Further, the oxidation process was optimized evaluating the effect of varying catalyst doses (50–300 mg L^−1^), hydrogen peroxide doses (12.5–62.5 mM), MB concentration (5–10 mg L^−1^), temperatures (20 – 70 °C), and pH (3–8). The catalytic activity of the recycled catalysts was also investigated, and the percentage of residual hydrogen peroxide was determined in each cycle. The concentration of MB in the samples was determined at 665 nm by HACH Lange DR-5000 UV-VIS spectrophotometer in a quartz cell with a path of 1 cm. The degradation efficacy of the heterogeneous Fenton process was determined using eqn (1).1
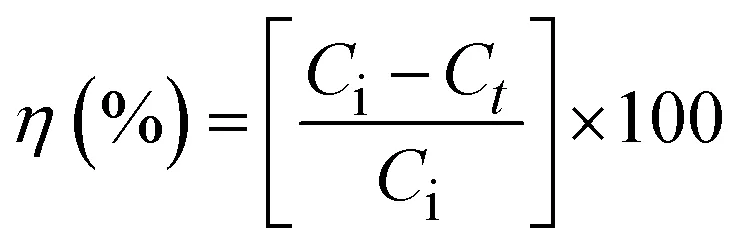
where: *η* = the removal efficiency of MB (%). *C*_i_ = initial concentration of MB. *C*_*t*_ = concentration of MB at time “*t*”.

The mineralization extent of the heterogeneous Fenton process was analyzed by determining the organic carbon content of the MB solutions. The samples were analyzed using a TOC-VCPN, Shimadzu analyzer (V-Series) with an auto sampler. First, the standard solutions with known concentrations of 2.5, 5.0, 10.0, and 20.0 mg C per L were prepared using potassium hydrogen phthalate stock solution. A calibration curve was obtained by analyzing the standard samples, and after each analysis the injection syringe was auto sparged with 2 M HCl. The samples were analyzed without dilution and using the same calibration curve to determine TOC present in each sample. The mineralization efficiency of the heterogeneous Fenton process was determined using eqn (2).2
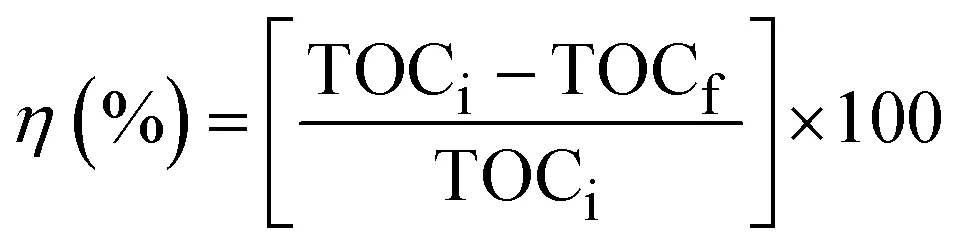
where: *η* = the TOC removal efficiency of the Fenton process (%). TOC_i_ = initial TOC value of the samples. TOC_f_ = final TOC value of the samples.

US EPA 3051 method using Inductivity Coupled Plasma-Atomic Emission Spectroscopy (ICP-AES vista pro) was used for the evaluation of the leaching of supported metals from the used catalysts during the heterogeneous Fenton process. First, a calibration curve was obtained by using an ICP 23-element standard solutions which were prepared in 5% HNO_3_. Yttrium (Y) was used as an internal standard. The method detection limit (MDL) was calculated as 3*s*/*m* (*s* = standard deviation of 10 replicate blanks and *m* = slope of the calibration curve for each element).

## Results and discussion

### Catalyst characterization

The composition and textural properties of the investigated catalysts are summarized in Table 1. All samples exhibit relatively low surface areas (<5 m^2^ g^−1^), which is consistent with the intrinsic characteristics of the Si_3_N_4_ support. The pristine silicon nitride shows a surface area of approximately 8 m^2^ g^−1^, while metal impregnation leads to a slight decrease due to partial pore blocking and coverage of the support surface by metal oxide species. Rietveld refinement of the XRD data confirms that the metal loading in all samples is close to the nominal value (5 wt%), indicating the effectiveness of the impregnation procedure.

**Table 1:** Composition and surface area of investigated samples

Sample	Nominal composition	M^a^ (wt%)	Surface area (m^2^ g^−1^)
Co/Si_3_N_4_	Co(5 wt%)/Si_3_N_4_	4.9	3
Cu/Si_3_N_4_	Cu(5 wt%)/Si_3_N_4_	4.9	2
Fe/Si_3_N_4_	Fe(5 wt%)/Si_3_N_4_	4.8	5
Zn/Si_3_N_4_	Zn(5 wt%)/Si_3_N_4_	5.0	2

a From Rietveld refinement.

The crystalline structure of the catalysts was investigated by powder X-ray diffraction (XRD). As shown in Fig. 1A, all diffraction patterns are dominated by reflections corresponding to the β-Si_3_N_4_ support, together with minor contributions from sinoite (Si_2_N_2_O, <2%). β-Si_3_N_4_ consists of layers of silicon and nitrogen atoms stacked along the [0001] direction, where each silicon atom occupies the center of a distorted tetrahedron coordinated by nitrogen atoms.^52^ This structure provides excellent chemical and thermal stability, making silicon nitride a promising support for catalytically active phases operating under harsh conditions.^53^ In addition to the support reflections, characteristic peaks corresponding to the oxide phases of the supported metals are observed. The Co/Si_3_N_4_ sample displays diffraction peaks at 2*θ* = 31.3° and 36.9°, which are attributed to the spinel Co_3_O_4_ phase crystallizing in the cubic *Fd*3̄*m* space group. For Cu/Si_3_N_4_, reflections located at 32.5°, 35.5°, and 38.7° are assigned to monoclinic CuO (*C*2/*c* space group). The Fe/Si_3_N_4_ catalyst exhibits diffraction peaks at 24.2°, 33.2°, 35.7°, and 54.2°, corresponding to hematite (α-Fe_2_O_3_, *R*3̄*c* space group). Finally, the Zn/Si_3_N_4_ sample shows characteristic reflections at 31.8°, 34.4°, and 56.6°, associated with the wurtzite structure of ZnO (*P*6_3_*mc* space group).

**Fig. 1:**
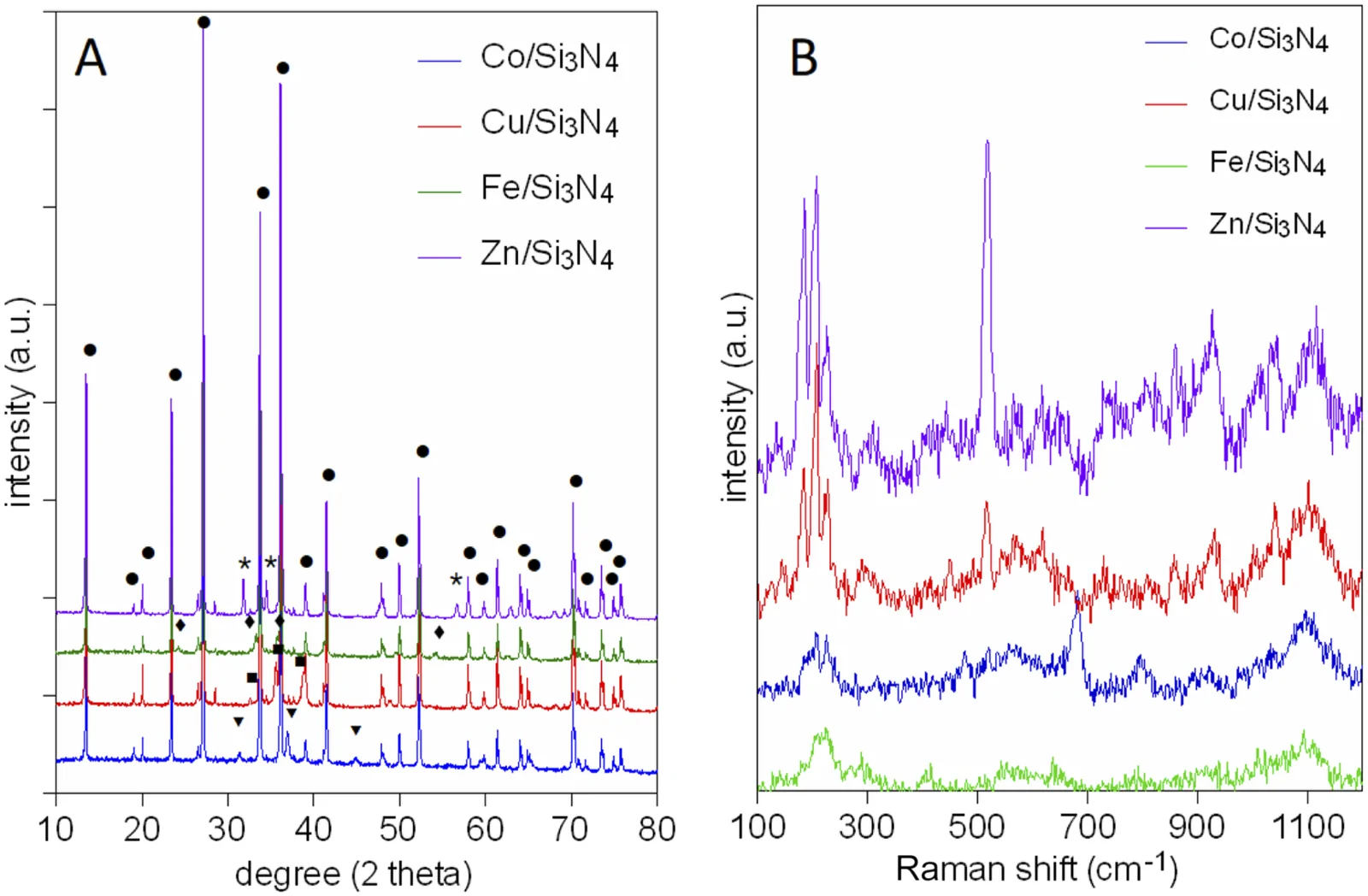
(A) XRD profiles of Si_3_N_4_ based catalysts (Si_3_N_4_, ●; Si_2_N_2_O, ○; Co_3_O_4_, ▼; CuO, ■; Fe_2_O_3_, ♦; ZnO, *); (B) Raman spectra of Si_3_N_4_ based catalysts.

These structural assignments are further supported by Raman spectroscopy (Fig. 1B and Table S1). The β-Si_3_N_4_ support exhibits sharp Raman bands in the 182–225 cm^−1^ region corresponding to lattice vibrational modes,^54,55^ along with additional bands between 400 and 1100 cm^−1^ attributed to internal structural vibrations. The presence of Co_3_O_4_ in the Co/Si_3_N_4_ catalyst is confirmed by Raman bands at approximately 470, 510, and 675 cm^−1^, which are characteristic of the spinel structure.^56^ The Cu/Si_3_N_4_ sample shows a broad spectral feature in the 280–320 cm^−1^ region, consistent with typical CuO vibrations observed at 288, 330, and 621 cm^−1^.^56,57^ In the case of Fe/Si_3_N_4_, the Raman spectrum exhibits characteristic hematite peaks at 146, 230, 293, and 412 cm^−1^.^58^ For Zn/Si_3_N_4_, the bands observed in the 400–500 and 700–800 cm^−1^ ranges confirm the presence of ZnO with wurtzite structure.^59^

Overall, these results demonstrate that all transition metals are present predominantly in oxide form after calcination. These oxide species are known to act as active redox centers capable of activating hydrogen peroxide and generating reactive oxygen species through redox cycling between different oxidation states.^29,60^ In the heterogeneous Fenton-like mechanism, the catalytic process involves the continuous transformation between M^*n*+^ and M^(*n*−1)+^ surface species (where M represents Co, Cu, Fe, or Zn), which promotes the formation of reactive oxygen species (ROS) involved in pollutant degradation (eqn (3) and (4)):3
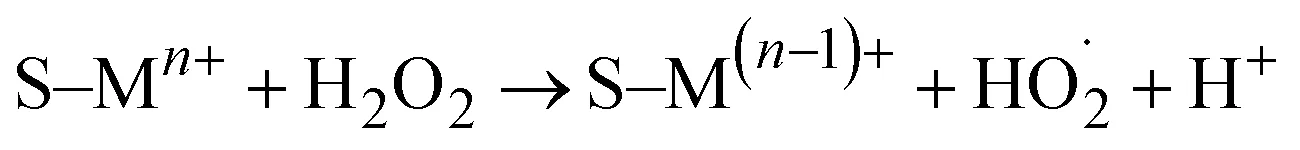

4 S–M^(*n*−1)+^ + H_2_O_2_ → S–M^*n*+^ + HO˙ + HO^−^

The M^*n*+^/M^(*n*−1)+^ redox cycle is crucial for the degradation and mineralization of organics and depends on parameters such as pH, temperature, catalyst loading, and oxidant concentration, which influence radical generation and catalytic efficiency.

The morphology and dispersion of the active phases were further examined by high-angle annular dark-field scanning transmission electron microscopy (HAADF-STEM) coupled with energy-dispersive X-ray spectroscopy (EDX). Fig. 2A shows a representative image of the pristine Si_3_N_4_ support, which consists of flake-like particles with sizes ranging from approximately 50 to 500 nm. For the Co/Si_3_N_4_ catalyst (Fig. 2B and C), spherical nanoparticles with diameters between 40 and 80 nm are observed to be well anchored on the Si_3_N_4_ surface.

**Fig. 2:**
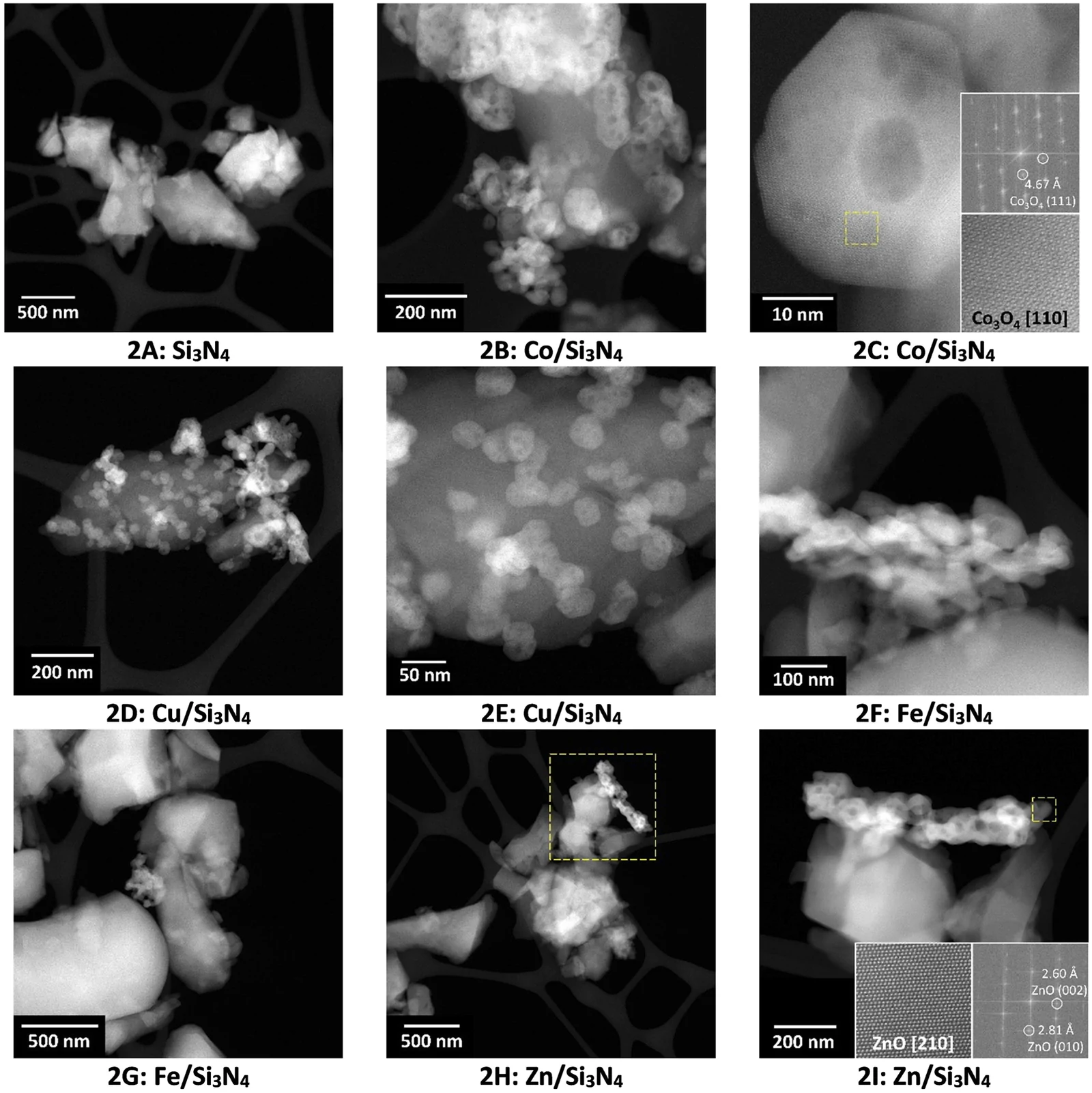
HAADF-STEM images of Si_3_N_4_ based catalysts.

High-resolution imaging reveals that these particles are highly crystalline and correspond to the Co_3_O_4_ spinel phase.

The particle in Fig. 2C has been selected to obtain EDX maps (Fig. 3A). As expected, the N and Si signals correspond to the Si_3_N_4_ support, and the Co signal coincides with the position of the particles with higher electron contrast. The particle is perfectly crystalline and it is oriented along the [110] crystallographic direction of the Co_3_O_4_ spinel structure. The inset in the figure shows the position of the atoms (area inside the yellow square) and the Fourier Transform image shows spots at 4.67 Å corresponding to the (111) crystallographic planes of the Co_3_O_4_ structure.

**Fig. 3:**
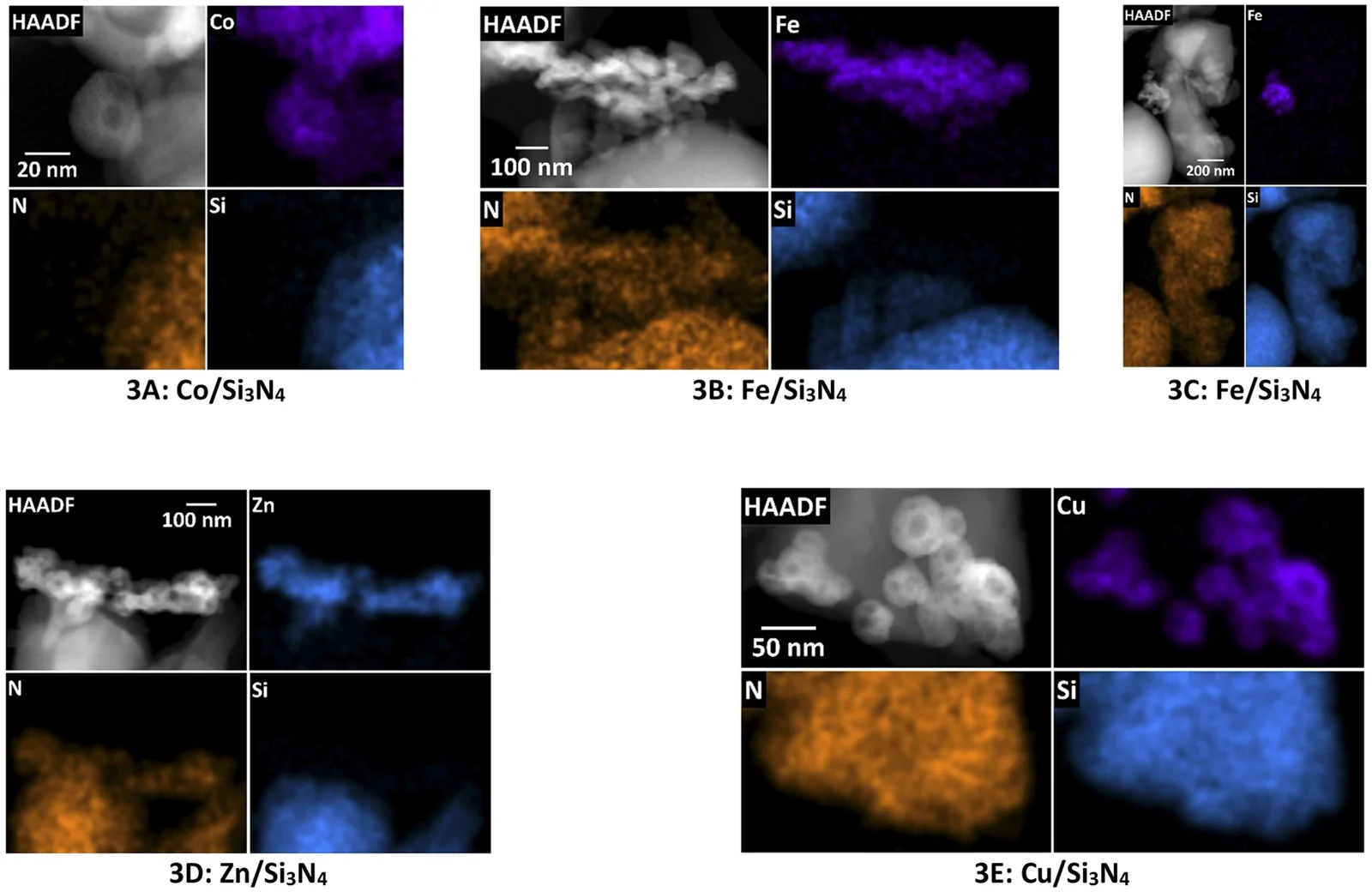
EDX maps of Si_3_N_4_ based catalysts.

Fig. 2D shows a representative image of Cu/Si_3_N_4_. Similarly, to the previous sample, there are spherical-type particles well dispersed over the Si_3_N_4_ support. In this case, the supported copper oxide particles are more finely dispersed, with average particle sizes ranging from 30 to 40 nm. A close-up view is depicted in Fig. 2E. Fig. 3E shows EDX maps of a selected area of the sample. As expected, the N and Si signals appear distributed over the whole area corresponding to the Si_3_N_4_ support, and the Cu signal match perfectly with the particles with higher electron contrast. EDX maps further confirm the homogeneous distribution of copper nanoparticles over the Si_3_N_4_ support. In contrast, the Fe-supported catalyst (Fig. 2F and G) shows a lower degree of dispersion. The iron oxide particles appear aggregated and irregularly distributed across the support surface, forming clusters rather than isolated nanoparticles. This reduced dispersion is consistent with the corresponding EDX maps, which show localized regions with strong Fe signals. They do not show any particular morphology and the particles tend to cluster. Fig. 3B and C show the EDX maps of the regions, corresponding to Fig. 2F and G, respectively, with Si and N signals corresponding to the Si_3_N_4_ support and the Fe signal matching perfectly with the particles with higher electron contrast. A similar behavior is observed for the Zn/Si_3_N_4_ catalyst (Fig. 2H). In this case, the ZnO particles tend to form agglomerated clusters composed of partially fused crystallites. The area inside the yellow square in Fig. 2H is shown at higher magnification in Fig. 2I. The particles occur as clumps of smaller particles which are partly fused with each other. The inset corresponds to the area marked inside the yellow square and shows that the particles correspond to the ZnO structure. The particle is oriented along the [210] crystallographic direction and the Fourier transform shows spots at 2.60 and 2.61 Å corresponding to the (002) and (010) crystallographic planes of ZnO, respectively. Fig. 3D shows EDX maps, where the Si and N signals match with the Si_3_N_4_ support and the Zn signal corresponds to the ZnO particles. Overall, the HAADF-STEM-EDX analysis reveals significant differences in metal dispersion depending on the nature of the supported metal. In particular, Cu/Si_3_N_4_ exhibits the smallest particle size and the most homogeneous dispersion, which is expected to enhance the availability of active surface sites and facilitate efficient redox cycling during the Fenton-like reaction.

### Catalytic results

#### Preliminary tests

Evaluating the adsorption contribution is of fundamental importance because the ultimate objective of the process is the mineralization of methylene blue, rather than its apparent removal solely through adsorption. Moreover, strong adsorption of the pollutant and/or its reaction intermediates on the catalyst surface may adversely affect catalytic performance by blocking or poisoning active sites. To clarify the contribution of non-catalytic pathways, a series of blank and control experiments was performed (Table 2S). The results show that MB removal in the absence of catalyst (MB + H_2_O_2_) remains limited (13% degradation; 7% TOC removal), indicating that direct oxidation by H_2_O_2_ is weak under the adopted conditions. Likewise, the system containing bare Si_3_N_4_ and H_2_O_2_ exhibits only modest activity (34% degradation; 9% TOC removal), confirming that the support alone does not significantly contribute to MB oxidation. Finally, the MB + M/Si_3_N_4_ trial shows negligible removal (5–7% degradation; 2–4% TOC), demonstrating that adsorption onto the catalyst surface is minimal. These results collectively confirm that the observed degradation and mineralization in the catalytic tests arise predominantly from heterogeneous Fenton-like oxidation, rather than from adsorption or non-catalytic H_2_O_2_ reactivity.

#### Effect of pH

The catalyst provides active sites for pollutant adsorption, followed by the generation of reactive oxygen species *via* redox cycling of the oxidant. These reactive radicals then attack and degrade the organic contaminants. The effectiveness of the heterogeneous Fenton process is strongly dependent on the pH of the reaction system.^61,62^ In general, acidic conditions are favorable for achieving optimal Fenton performance.^63^ However, the ideal pH is catalyst-specific and often aligns with the point of zero charge (PZC) to maximize catalytic efficiency.^29,64^ The pH plays a crucial role for several reasons: it governs both the stability of the catalyst and of hydrogen peroxide,^65^ and it also influences the temporary adsorption of pollutants on the catalyst surface.^15^

Therefore, the catalytic performance of the investigated M/Si_3_N_4_ systems was evaluated over a pH range between 3 and 8 in order to assess their activity under both acidic and near-neutral conditions. While MB discoloration served as a preliminary indicator of activity, Total Organic Carbon (TOC) removal was employed as the definitive criterion for process optimization. The influence of initial pH on the degradation of Methylene Blue (MB) was evaluated by monitoring the concentration changes over a 30-minute reaction period. The kinetic profiles for the M/Si_3_N_4_ (M = Cu, Fe, Co, Zn) catalysts are shown in Fig. 4A. Across all catalysts, a characteristic “burst effect” is observed within the first 5 minutes of the reaction at optimal pH values (3–5). During this initial stage, the MB degradation efficiency reaches a plateau of nearly 100%. This rapid kinetics suggests a high surface reactivity. The Si_3_N_4_ support facilitates a high dispersion of active metal sites, allowing for instantaneous H_2_O_2_ activation upon contact. In addition, this effect can be correlated to an efficient radical generation; the rapid discoloration indicates that the generation of ROS occurs at a rate significantly higher than their scavenging by secondary reactions in the early stages.

**Fig. 4:**
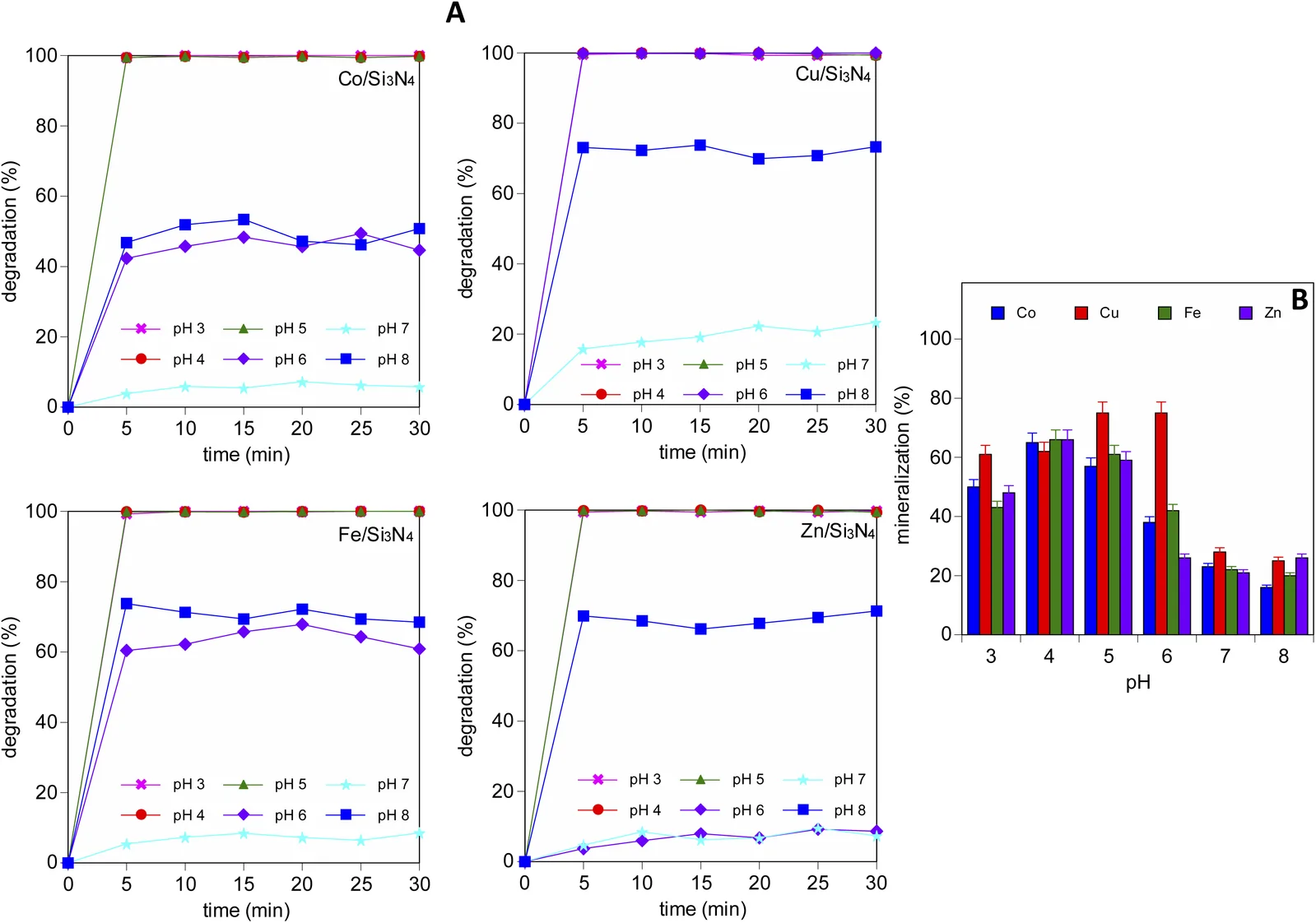
Effect of pH on (A) MB degradation and (B) MB mineralization (reaction conditions: catalyst dose 150 mg L^−1^ for Cu/Si_3_N_4_ and 300 mg L^−1^ for Co/Si_3_N_4_, Fe/Si_3_N_4_ and Zn/Si_3_N_4_; H_2_O_2_ dose 50 mM; temperature 70 °C; MB 10 mg L^−1^; reaction time 30 minutes).

A detailed comparison of the kinetic plots reveals a distinct superiority of the Cu/Si_3_N_4_ system. This catalyst maintains an ultra-fast kinetic profile (reaching 100% in <5 min) across a remarkably wide range from pH 3 to 6. In contrast, the Fe, Co, and Zn analogs show a dramatic decline in the slope of the kinetic curves as the pH increases from 5 to 6. For Fe, Co, and Zn, pH 6 serves as a critical threshold where the reaction transitions from a diffusion-controlled regime to a reaction-limited regime, with degradation efficiencies dropping to 60%, 50%, and 10% respectively. As the system moves toward alkaline conditions (pH 7–8), the kinetic curves for all catalysts (except Cu at pH 8) become significantly flatter. This kinetic inhibition can be attributed to the oxidant depletion and to the surface deactivation. Indeed, the accelerated, non-productive decomposition of H_2_O_2_ into water and oxygen, reduces the steady-state concentration of reactive ˙OH/ROS species, while the potential formation of metal hydroxide layers on the catalyst surface at higher pH may block the active redox sites, hindering the adsorption of MB molecules. The mineralization data (TOC removal) provides a more rigorous assessment of the catalytic process than simple discoloration (Fig. 4B). For Cu/Si_3_N_4_ system the highest TOC removal (75%) was observed at near-neutral pH (5–6), corresponding to broader catalytic activity in terms of both MB degradation and mineralization. This broad operating window is significant as it minimizes metal leaching, thereby enhancing recyclability and overall process sustainability. The improved performance may be associated with the redox properties of CuO and, potentially, with Cu^2+^/Cu^+^ electron-transfer processes, which could facilitate continuous redox cycling and contribute to the generation of reactive oxygen species even under less acidic conditions. Furthermore, the slightly acidic nature of the degradation by-products helps maintain a low risk of metal ion release. Conversely, performance was sharply suppressed at pH 7–8, likely due to the accelerated decomposition of H_2_O_2_ into water and oxygen, which depletes the available oxidant.^66,67^ Fe/Si_3_N_4_, Zn/Si_3_N_4_, and Co/Si_3_N_4_ reach maximum TOC removal at pH 4. At higher pH, both degradation and mineralization declined, likely due to reduced reactive oxygen species generation, lower oxidation capacity of ROS,^68^ and H_2_O_2_ decomposition.^69^ Optimal pH also helps prevent secondary pollution from metal leaching. At pH 7, Zn/Si_3_N_4_ and Co/Si_3_N_4_ show a “mineralization–discoloration paradox” where TOC removal (20–25%) exceeds MB degradation (<10%). This suggests that at neutral pH, these metals promote a pathway that partially mineralizes the organic load while leaving the parent chromophore (or light-absorbing fragments) intact, potentially due to different radical-attack site preferences compared to acidic conditions. Overall, these results demonstrate that the nature of the supported metal plays a key role in determining the pH tolerance of the catalyst, with Cu/Si_3_N_4_ showing the most promising behavior for potential applications under realistic wastewater treatment conditions.

The kinetic stability of the catalysts was qualitatively assessed by analyzing the initial degradation rates, calculated from the slopes of the concentration profiles during the first 5 min of reaction. At pH 4, all catalysts exhibited comparable kinetic behavior, characterized by very steep initial slopes that indicate rapid pollutant degradation and efficient hydrogen peroxide activation. However, increasing the pH to 6 resulted in a clear kinetic divergence. The Cu/Si_3_N_4_ catalyst preserved nearly unchanged initial reaction rates, maintaining a rapid degradation regime with complete MB removal within less than 5 min across the pH range 3–6. In contrast, the Fe/Si_3_N_4_, Co/Si_3_N_4_, and Zn/Si_3_N_4_ catalysts showed a pronounced decrease, indicating a significant loss of catalytic efficiency at near-neutral pH. This behavior highlights the key role of the metal–support interaction in controlling the catalytic stability. The superior kinetic stability of the Cu/Si_3_N_4_ catalyst suggests that the interaction between copper species and the Si_3_N_4_ support plays a relevant role in preserving catalytic activity at near-neutral pH. The nitrogen-rich surface could contribute to stabilizing the active copper sites and maintaining their catalytic functionality during the reaction.

#### Effect of catalyst dose

The optimization of catalyst dosage is a pivotal factor in heterogeneous Fenton-like systems, balancing catalytic efficiency against economic viability and the potential for secondary pollution due to metal leaching or radical scavenging. The influence of catalyst concentration (ranging from 50 to 300 mg L^−1^) on MB degradation kinetics and TOC mineralization is presented in Fig. 5. As shown in Fig. 5A, all M/Si_3_N_4_ catalysts achieved complete MB discoloration within 5–10 minutes, regardless of the dosage. Interestingly, the kinetic curves are largely overlapping, particularly for Fe, Co, and Zn systems. This suggests that for primary dye degradation (discoloration), even the lowest dosage (50 mg L^−1^) provides sufficient active sites to handle the initial pollutant load under the tested conditions. However, the true impact of the dosage is revealed through the mineralization (TOC) data (Fig. 5B). The mineralization efficiency exhibited two distinct behaviors depending on the metal species. For Fe, Zn, and Co/Si_3_N_4_ TOC removal showed a linear correlation with dosage, increasing from approximately 52–55% at 50 mg L^−1^ to 65–66% at 300 mg L^−1^. This trend is attributed to the increased availability of surface active sites, which facilitates a higher rate of H_2_O_2_ homolysis and, consequently, a greater density of ROS capable of attacking recalcitrant intermediate products.^70,71^ Cu/Si_3_N_4_ consistently outperformed the other catalysts across the entire dosage range. Maximum mineralization (80%) was achieved at a relatively low dosage of 150 mg L^−1^. Beyond this threshold, a slight decline in TOC removal (to 75%) was observed. This “optimum point” behavior suggests two possible phenomena: (a) particle agglomeration, at higher concentrations, the hydrophilicity of the Cu/Si_3_N_4_ particles may lead to aggregation, effectively reducing the accessible active surface area per unit mass; (b) radical scavenging, an excess of active copper sites may trigger the scavenging of radicals by the catalyst itself (*e.g.* Cu^+^ + ˙OH → Cu^2+^ + OH^−^), or lead to the consumption of ROS by H_2_O_2_ in unproductive side reactions, thereby suppressing the overall mineralization efficiency.^72^ The remarkable performance of Cu/Si_3_N_4_ achieving 80% TOC removal at a dosage of only 150 mg L^−1^, stands in stark contrast to the other catalysts, which failed to reach comparable mineralization levels even at twice the concentration (300 mg L^−1^).

**Fig. 5:**
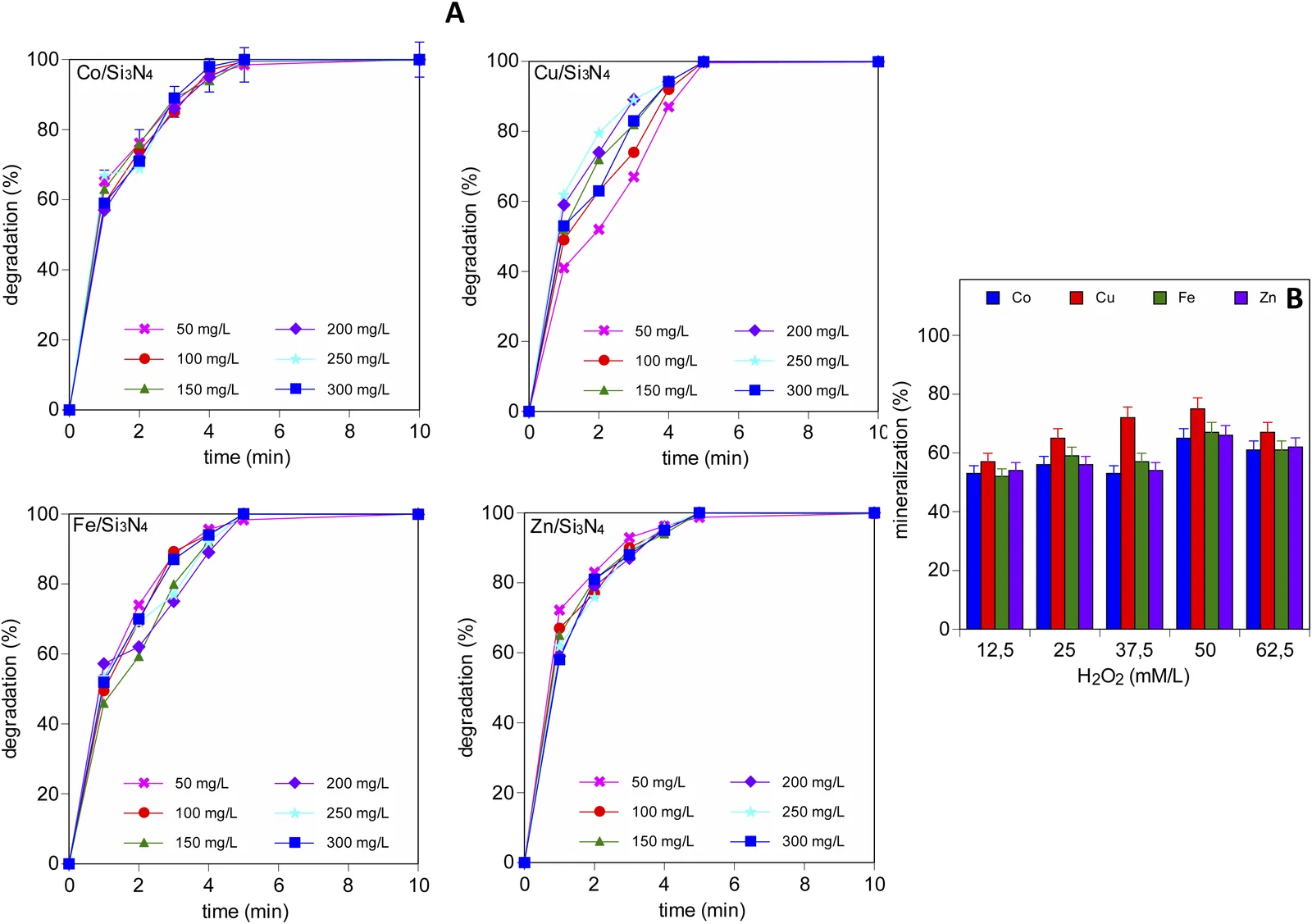
Effect of catalyst dose on (A) MB degradation and (B) MB mineralization (reaction conditions: pH 6 for Cu/Si_3_N_4_ and pH 4 for Co/Si_3_N_4_, Fe/Si_3_N_4_ and Zn/Si_3_N_4_; H_2_O_2_ dose 50 mM; temperature 70 °C; MB 10 mg L^−1^; reaction time 30 minutes).

This disparity underscores the exceptional intrinsic activity of the copper-nitride synergistic sites, which likely facilitate a more efficient utilization of generated Reactive Oxygen Species. This allows for a reduction in the required catalyst mass, which is a key advantage for minimizing operational costs and sludge production in practical wastewater treatment. In addition, reduced secondary pollution lowering the total metal load in the system, thereby mitigating potential metal leaching. Therefore, the Cu/Si_3_N_4_ system at 150 mg L^−1^ represents the optimal balance between catalytic performance and material economy, making it a superior candidate for scaled-up wastewater remediation.

#### Effect of oxidant dose

The oxidant plays a critical role in the heterogeneous Fenton process as the source of free radicals.^73^ An effective catalyst must be highly active, stable, and capable of efficiently converting H_2_O_2_ into ˙OH/ROS radicals.^74^ The generation of ˙OH/ROS depends on the ability of transition metals on the catalyst surface to cycle between multiple oxidation states, producing radicals that oxidize organic pollutants. The optimal oxidant dose depends on the type of transition metal and the reaction pH and must be carefully controlled. Insufficient H_2_O_2_ limits degradation, while excess increases costs and may induce oxidative stress in aquatic environments.^3^ The kinetic plots (Fig. 6A) show that complete MB discoloration (Fig. 2S) occurs rapidly (within 5 minutes) for all catalysts at all tested dosages (12.5–62.5 mM). This suggests that the primary oxidation of the parent dye molecule is relatively insensitive to H_2_O_2_ concentration within this range. However, the TOC mineralization efficiency (Fig. 6B) reveals a much more nuanced dependency. Mineralization increases progressively as the H_2_O_2_ dose rises from 12.5 to 50 mM. At 50 mM, Cu/Si_3_N_4_ reaches its peak efficiency (approx. 75% TOC removal), significantly outperforming the Fe, Co, and Zn catalyst (approx. 60–65%). This trend is expected, as a higher oxidant concentration provides more precursors for radical generation *via* metal-centered redox cycling. A notable decline in mineralization is observed for all catalysts when the dosage is increased to 62.5 mM. This phenomenon is a classic example of radical scavenging by the excess oxidant itself, governed by the following reaction:˙OH + H_2_O_2_ → HOO˙ + H_2_O

**Fig. 6:**
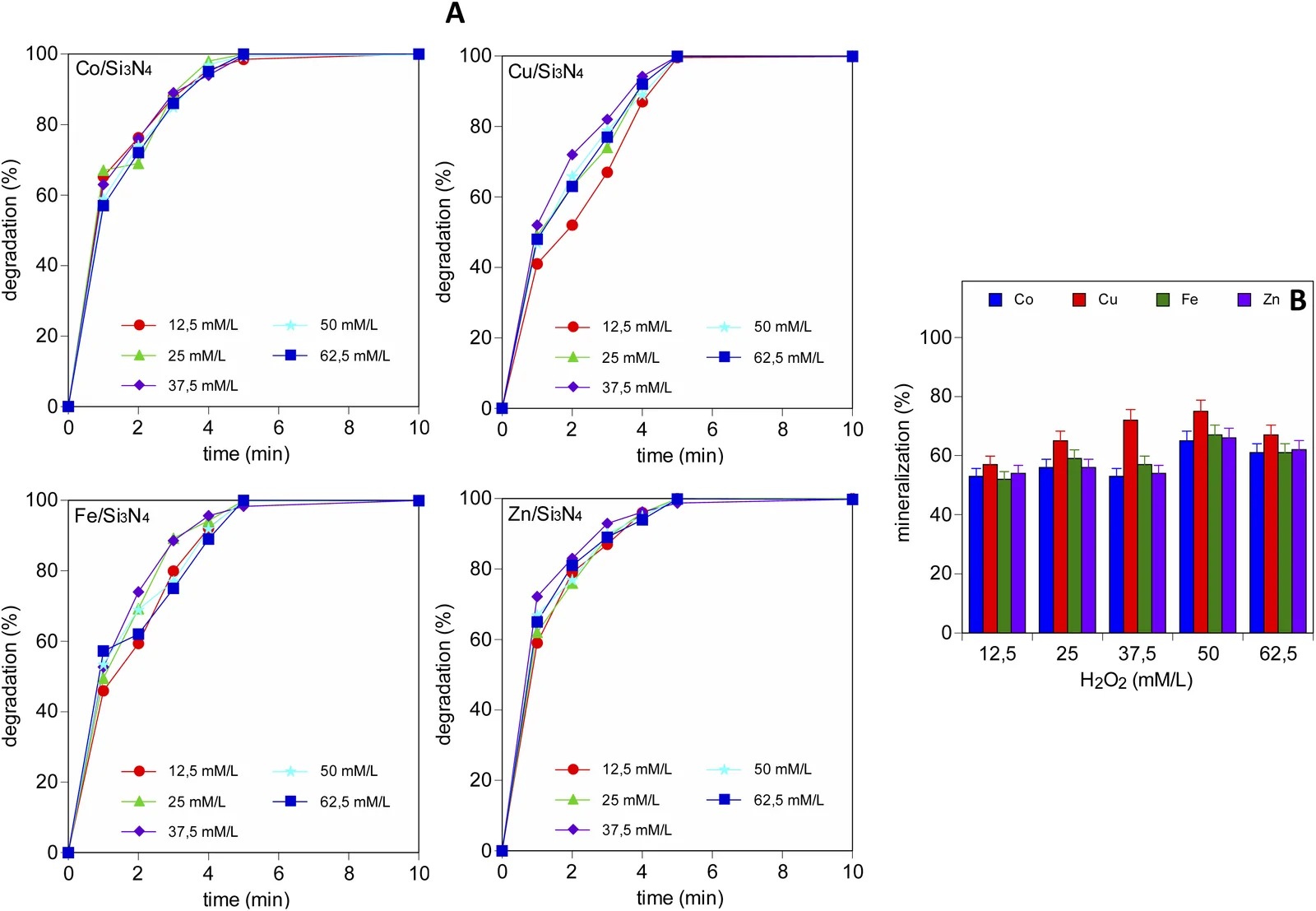
Effect of H_2_O_2_ dose on (A) MB degradation and (B) MB mineralization (reaction conditions: pH 6 for Cu/Si_3_N_4_ and pH 4 for Co/Si_3_N_4_, Fe/Si_3_N_4_ and Zn/Si_3_N_4_; catalyst dose 150 mg L^−1^ for Cu/Si_3_N_4_ and 300 mg L^−1^ for Co/Si_3_N_4_, Fe/Si_3_N_4_ and Zn/Si_3_N_4_; temperature 70 °C; MB 10 mg L^−1^; reaction time 30 minutes).

This reaction is detrimental because it consumes high-energy ˙OH radicals (*E*^0^ = 2.80 V)^75^ and generates hydroperoxyl radicals (HOO˙) which possess a significantly lower oxidation potential (*E*^0^ = 1.70 V), making them less effective at breaking down recalcitrant organic intermediates.^76^ Furthermore, Cu/Si_3_N_4_ maintains a higher mineralization baseline even at sub-optimal dosages (*e.g.*, at 12.5 mM, Cu reaches ∼58% TOC removal while others stay below 55%).

This suggests that the copper-nitride synergistic sites are more oxidant-efficient, meaning they can generate a sufficient radical flux even when the precursor concentration is low.

#### Effect of MB concentration

The initial concentration of the pollutant is a key factor in determining the practical applicability of the heterogeneous Fenton process, as it influences the molar ratio between the oxidant, the active sites, and the target molecules. To evaluate the scalability of the process, the effect of initial MB concentration was investigated (Fig. 7). Unlike traditional Fenton systems where high pollutant loads typically suppress efficiency due to site saturation, the M/Si_3_N_4_ series exhibited remarkable resilience. As shown in the kinetic plots (Fig. 7A), all catalysts maintained an exceptionally high degradation rate, achieving nearly 100% discoloration within 5 minutes regardless of the initial dye concentration. The kinetic profiles for MB degradation remained nearly identical across the 5.0–10.0 mg L^−1^ range, indicating that the radical generation rate is not a limiting factor at these concentrations.

**Fig. 7:**
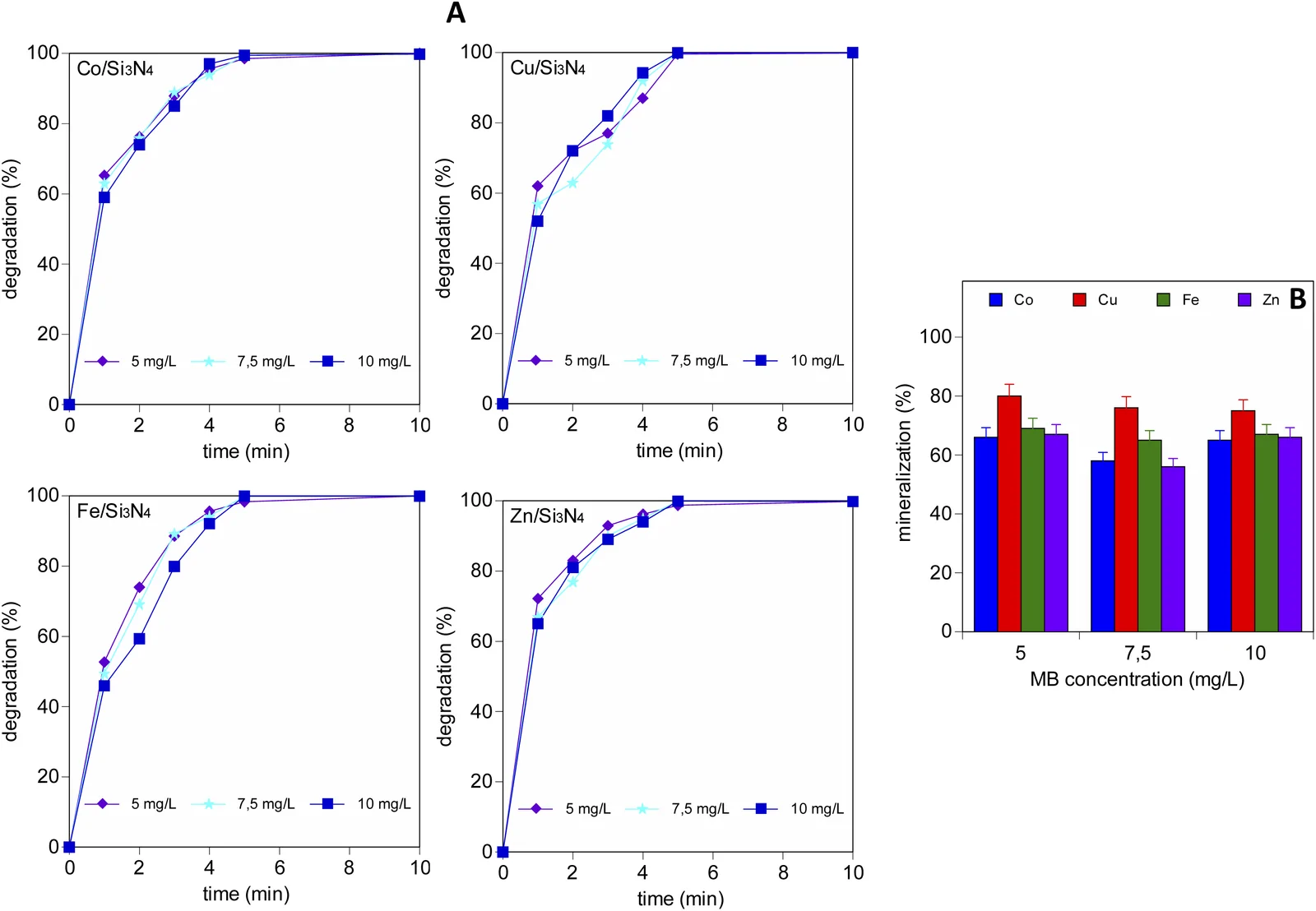
Effect of initial MB concentration on (A) MB degradation and (B) MB mineralization (reaction conditions: pH 6 for Cu/Si_3_N_4_ and pH 4 for Co/Si_3_N_4_, Fe/Si_3_N_4_ and Zn/Si_3_N_4_; catalyst dose 150 mg L^−1^ for Cu/Si_3_N_4_ and 300 mg L^−1^ for Co/Si_3_N_4_, Fe/Si_3_N_4_ and Zn/Si_3_N_4_; H_2_O_2_ dose 50 mM; temperature 70 °C; reaction time 30 minutes).

This stability suggests that the Si_3_N_4_ support suppresses the competitive inhibition commonly observed in Fenton processes, where high concentrations of organic molecules adsorb on the catalyst surface and block access to active sites. The TOC mineralization results (Fig. 7B) further confirm the robustness of the system. Indeed, TOC mineralization remained largely unaffected, with Cu/Si_3_N_4_ consistently achieving ∼75–80% removal. This suggests that the copper-nitride synergistic sites generate a radical flux sufficient to cleave the chromophore and mineralize the resulting intermediates even when the organic load is doubled. These results demonstrate that the catalysts possess a high capacity for handling varying organic loads, which is a crucial requirement for real-world wastewater treatment applications.

#### Effect of temperature

The heterogeneous Fenton process is strongly influenced by temperature, with increasing temperature generally leading to faster reaction kinetics.^77^ Although heterogeneous catalysts lower the activation energy, a minimum energy input is still required to overcome the reaction barrier and form products.^78^ Temperature is a key thermodynamic factor that influences the collision frequency between reactants and the catalyst surface, as well as the rate of radical generation.^79^ The results presented in Fig. 8 demonstrate a clear positive correlation between thermal input and catalytic efficiency. The M/Si_3_N_4_ catalytic system was evaluated over a temperature range of 20 °C to 70 °C (Fig. 8).

**Fig. 8:**
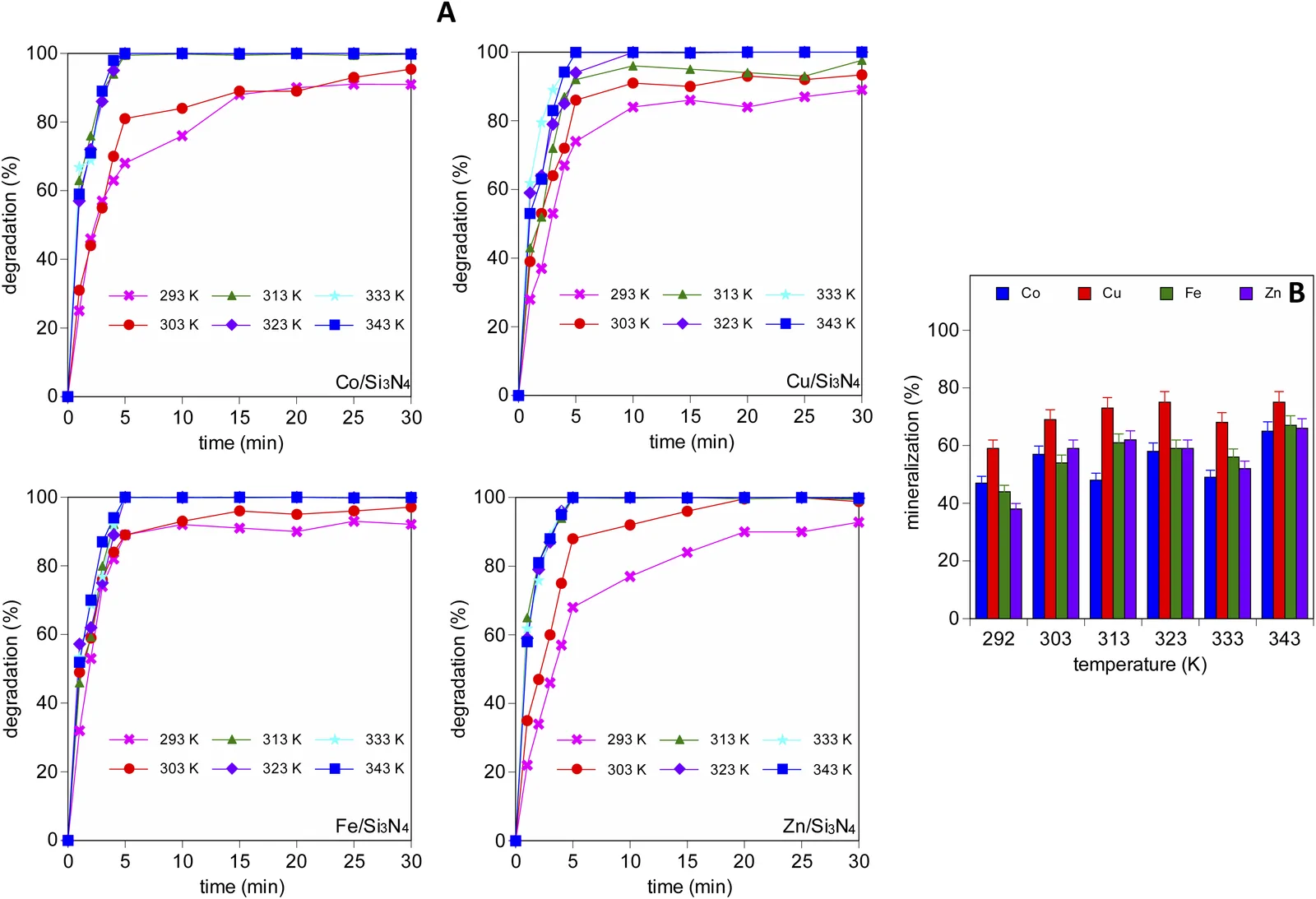
Effect of temperature on (A) MB degradation and (B) MB mineralization (reaction conditions: pH 6 for Cu/Si_3_N_4_ and pH 4 for Co/Si_3_N_4_, Fe/Si_3_N_4_ and Zn/Si_3_N_4_; catalyst dose 150 mg L^−1^ for Cu/Si_3_N_4_ and 300 mg L^−1^ for Co/Si_3_N_4_, Fe/Si_3_N_4_ and Zn/Si_3_N_4_; H_2_O_2_ dose 50 mM; MB 10 mg L^−1^; reaction time 30 minutes).

At the lowest tested temperature (20 °C), the system exhibits limited activity, particularly in terms of mineralization. However, increasing the temperature to 70 °C led to a significant enhancement in performance, with all catalysts reaching complete MB degradation (Fig. 8A) and TOC mineralization values between 65% and 75% (Fig. 8B). This temperature dependence is consistent with thermally accelerated reaction kinetics and may be associated with enhanced H_2_O_2_ activation and reactive oxygen species generation, thereby facilitating the degradation of MB and its more recalcitrant oxidation intermediates.^70^ Although an operating temperature of 70 °C might initially appear energetically demanding for conventional wastewater treatments, it may be compatible with closely mirrors real-world industrial scenarios. Textile wet processing operations naturally discharge hot, highly contaminated effluents.^80^ Consequently, the observed temperature dependence suggests that the process could benefit kinetically from the residual thermal energy of hot industrial effluents, potentially reducing the external energy demand associated with heating the wastewater. Further process-level energy and techno-economic assessments would, however, be required to quantify this potential benefit.

When integrated with the results from previous sections, a clear performance hierarchy emerges. pH dictates the feasibility of the reaction, while temperature and oxidant/catalyst dosages act as fine-tuning parameters for mineralization. A clear divergence in pH-dependent performance was observed: while the Co-, Fe-, and Zn/Si_3_N_4_ catalysts were restricted to a narrow acidic window (pH 5), the Cu/Si_3_N_4_ system exhibited remarkable resilience, maintaining high catalytic activity up to pH 6.

The mineralization data further corroborated this trend. Peak TOC removal for Co, Fe, and Zn systems occurred at pH 4, whereas Cu/Si_3_N_4_ achieved its maximum efficiency at pH 6. This shift toward near-neutrality is a significant finding, as it highlights the enhanced sustainability of the copper-based catalyst, reducing the need for intensive pH adjustment and minimizing the risk of metal leaching. The influence of other parameters can be summarized as follows: MB degradation was largely insensitive to dosage variations; however, mineralization was more responsive. For most catalysts, higher loadings (250–300 mg L^−1^) yielded marginal improvements, but Cu/Si_3_N_4_ demonstrated an optimal efficiency plateau at intermediate doses (150–200 mg L^−1^), beyond which performance stabilized or slightly declined due to scavenging effects. Similarly, increasing H_2_O_2_ concentration from 12.5 to 50 mM linearly enhanced mineralization by expanding the radical pool.

Elevated reaction temperatures acted as a kinetic accelerator, promoting both discoloration and mineralization through enhanced H_2_O_2_ homolysis. In contrast, variations in the initial MB concentration exerted only a minor influence on process efficiency, highlighting the strong catalytic robustness of the M/Si_3_N_4_ series and its potential for treating industrial effluents with variable organic loads.

#### Heterogeneous Fenton potential (HFP) of catalysts

In order to provide a more comprehensive evaluation of the catalytic performance, a new parameter termed Heterogeneous Fenton Potential (HFP) was introduced (eqn (5)):5
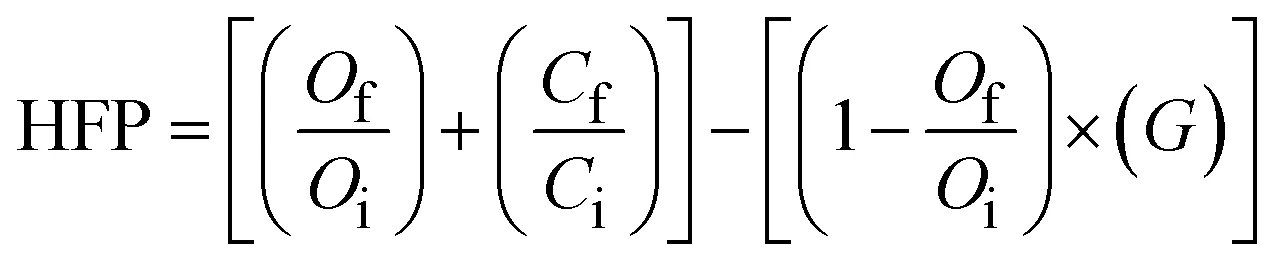
where: *O*_f_/*O*_i_ = fraction of oxidant utilized in Fenton oxidation. *C*_f_/*C*_i_ = fractional removal of TOC. 1 − *O*_f_/*O*_i_ = residual fraction of oxidant. *G* = difference between utilized oxidant and mineralized TOC.

The Heterogeneous Fenton Potential (HFP) is proposed as an empirical composite index that combines hydrogen peroxide consumption with the extent of TOC removal, providing an integrated descriptor of catalyst performance under the investigated reaction conditions. HFP is therefore not intended to represent the direct conversion of H_2_O_2_ into reactive radicals, since H_2_O_2_ may undergo non-productive decomposition and other parallel pathways. Instead, HFP descriptor serves as a practical integrated screening tool to evaluate catalyst performance by coupling oxidant depletion with mineralization efficiency.

Conventional catalytic assessments are commonly based on pollutant degradation efficiency and/or mineralization rates. Although these parameters provide important information on catalytic activity, they do not account for the amount of oxidant consumed to achieve the observed degree of mineralization. In heterogeneous Fenton-like systems, H_2_O_2_ can follow multiple reaction pathways. Consequently, extensive H_2_O_2_ consumption does not necessarily imply a correspondingly high production of reactive species or enhanced mineralization. The HFP was therefore introduced to consider, within a single empirical descriptor, both H_2_O_2_ consumption and the achieved degree of mineralization, enabling a comparative evaluation of catalyst performance under otherwise identical reaction conditions.

Higher HFP values indicate a more favorable balance between oxidant consumption and TOC removal according to the proposed empirical metric, whereas lower values indicate a less favorable balance between these two parameters. The HFP should therefore be interpreted as a comparative performance descriptor rather than as a direct measure of radical generation, H_2_O_2_-to-radical conversion, or reaction mechanism. Based on the mathematical definition of eqn (5), the theoretical upper limit of HFP is 2.0, corresponding to the simultaneous maximization of the terms describing oxidant utilization and TOC removal. This upper limit represents an idealized reference condition rather than a mechanistic or stoichiometric measure of reaction efficiency. Deviations from this value indicate a less favorable balance between oxidant consumption and mineralization within the proposed empirical framework. As shown in Fig. 9 and Table 3S, Cu/Si_3_N_4_ exhibited the highest HFP (1.71), consistent with its high mineralization efficiency (Fig. 4) and comparatively low residual H_2_O_2_ fraction (Fig. 6).

**Fig. 9:**
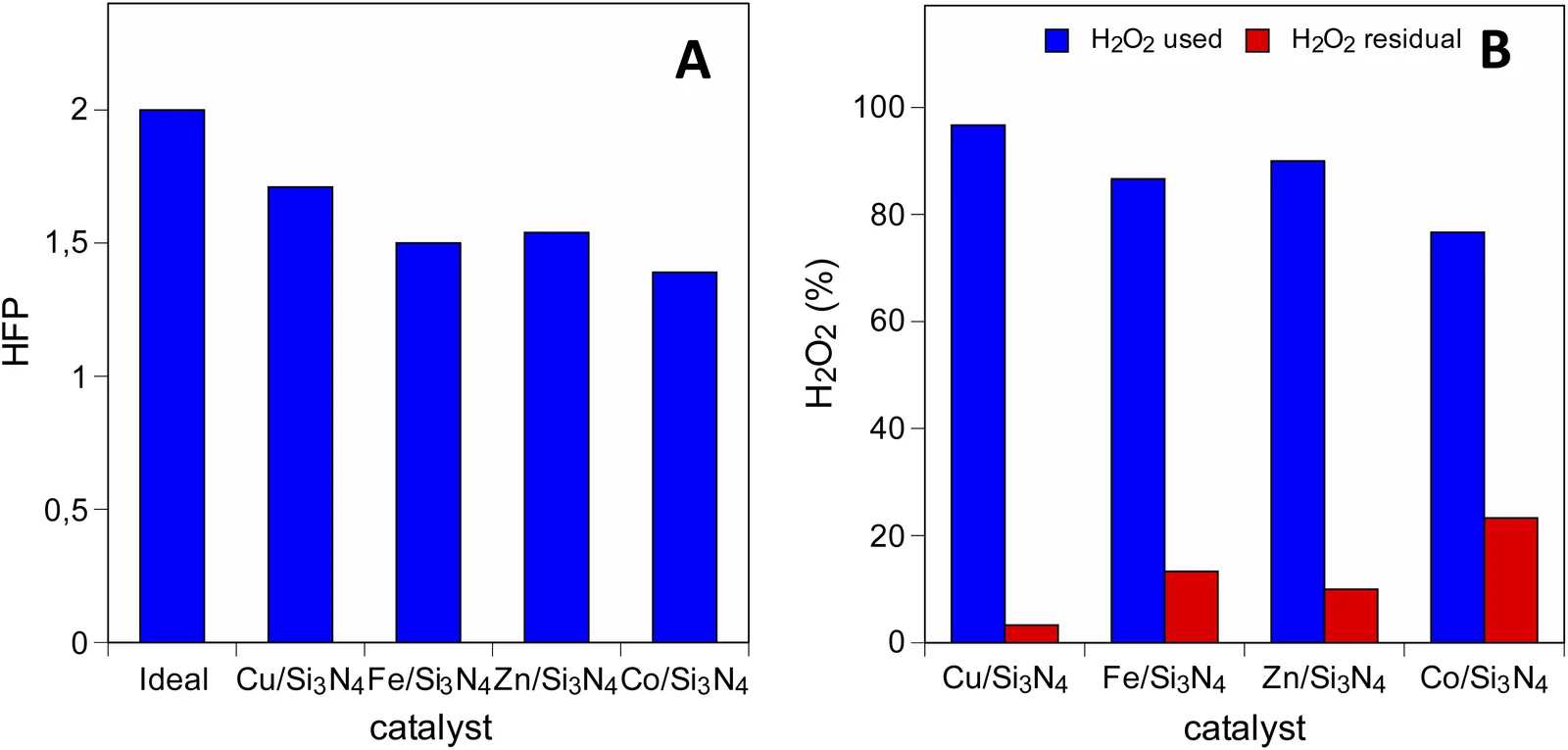
(A) The HFP of employed catalysts under optimal conditions. (B) Consumed and residual H_2_O_2_.

Fe/Si_3_N_4_ and Zn/Si_3_N_4_ showed HFPs of 1.50 and 1.54, respectively, indicating a less favorable balance between oxidant consumption and TOC removal compared with Cu/Si_3_N_4_. These results suggest that optimization of the initial H_2_O_2_ dosage could be investigated for these systems to further improve oxidant utilization and process economy. In contrast, Co/Si_3_N_4_ displayed the lowest HFP (1.39), despite a mineralization performance comparable to that of the other catalysts.

The analysis of the residual H_2_O_2_ fraction (Fig. 9B) provides additional information for interpreting the HFP. Cu/Si_3_N_4_ exhibited the lowest residual H_2_O_2_ (3%), indicating that nearly the entire oxidant was effectively utilized during the reaction. Fe/Si_3_N_4_ and Zn/Si_3_N_4_ showed moderate residual fractions (13% and 10%, respectively), indicating that the initial H_2_O_2_ dosage could be further refined without necessarily affecting the overall mineralization efficiency. In contrast, Co/Si_3_N_4_ displayed a substantially higher residual H_2_O_2_ (23%), confirming that the system operates under oxidant-excess conditions where part of the reagent remains unconsumed at the end of the reaction. This observation is consistent with its lower HFP value and suggests that the oxidant dosage could potentially be optimized to reduce residual H_2_O_2_. From a process-engineering perspective, the HFP can nevertheless provide a useful preliminary indication of whether the oxidant dosage is appropriately matched to the mineralization achieved. A relatively low HFP value identifies systems in which the balance between H_2_O_2_ consumption and TOC removal is less favorable and therefore highlights opportunities for optimization of the oxidant dosage and reaction conditions. In this context, the HFP can support comparative catalyst screening and preliminary assessment of reagent utilization, while more detailed mechanistic investigations would be required to determine the specific pathways responsible for H_2_O_2_ consumption. Overall, by combining oxidant consumption with mineralization data, the HFP provides an empirical descriptor that complements conventional degradation and mineralization metrics and enables a more comprehensive comparison of heterogeneous Fenton catalysts under otherwise identical experimental conditions.

### Reaction kinetics of MB degradation

Degradation follows apparent first-order kinetics for all catalysts (Fig. 1S), indicating that, under the investigated experimental conditions, the MB degradation rate can be adequately described by a pseudo-first-order kinetic model.^81,82^ The heterogeneous Fenton reactions proceeded extremely rapidly, as confirmed by the high rate constants (Fig. 1S). Nearly complete degradation of methylene blue (MB) was achieved within 5 minutes for all catalysts, as visually evidenced by the rapid discoloration of the solution (Fig. 2S). After 5 minutes, 90% degradation and 80% of maximum mineralization were achieved, with minimal improvement at longer times, suggesting rapid oxidation but incomplete mineralization due to the formation of more stable intermediates.

Among all catalysts, the Cu-based system demonstrated the highest mineralization efficiency under varying pH, temperature, oxidant and catalyst doses, MB concentration, and reaction time. This superior activity is attributed to the high dispersion of copper species and their small crystallite size (30–40 nm), as revealed by HAADF-STEM and XRD analyses. HAADF-STEM images showed uniformly distributed, finely dispersed CuO nanoparticles, and EDX mapping confirmed homogeneous elemental distribution. The enhanced dispersion increases the number of accessible active sites and facilitates efficient electron transfer, leading to improved degradation and mineralization performance. The temperature dependence of the apparent rate constants was further investigated using the Arrhenius relationship (eqn (7S)). Plots of ln *k vs.* 1/*T* were linear for all catalysts (*R*^2^ = 0.999, Fig. 10), yielding apparent activation energies (*E*_a_) of approximately 80 kJ mol^−1^ (Fig. 10).

**Fig. 10:**
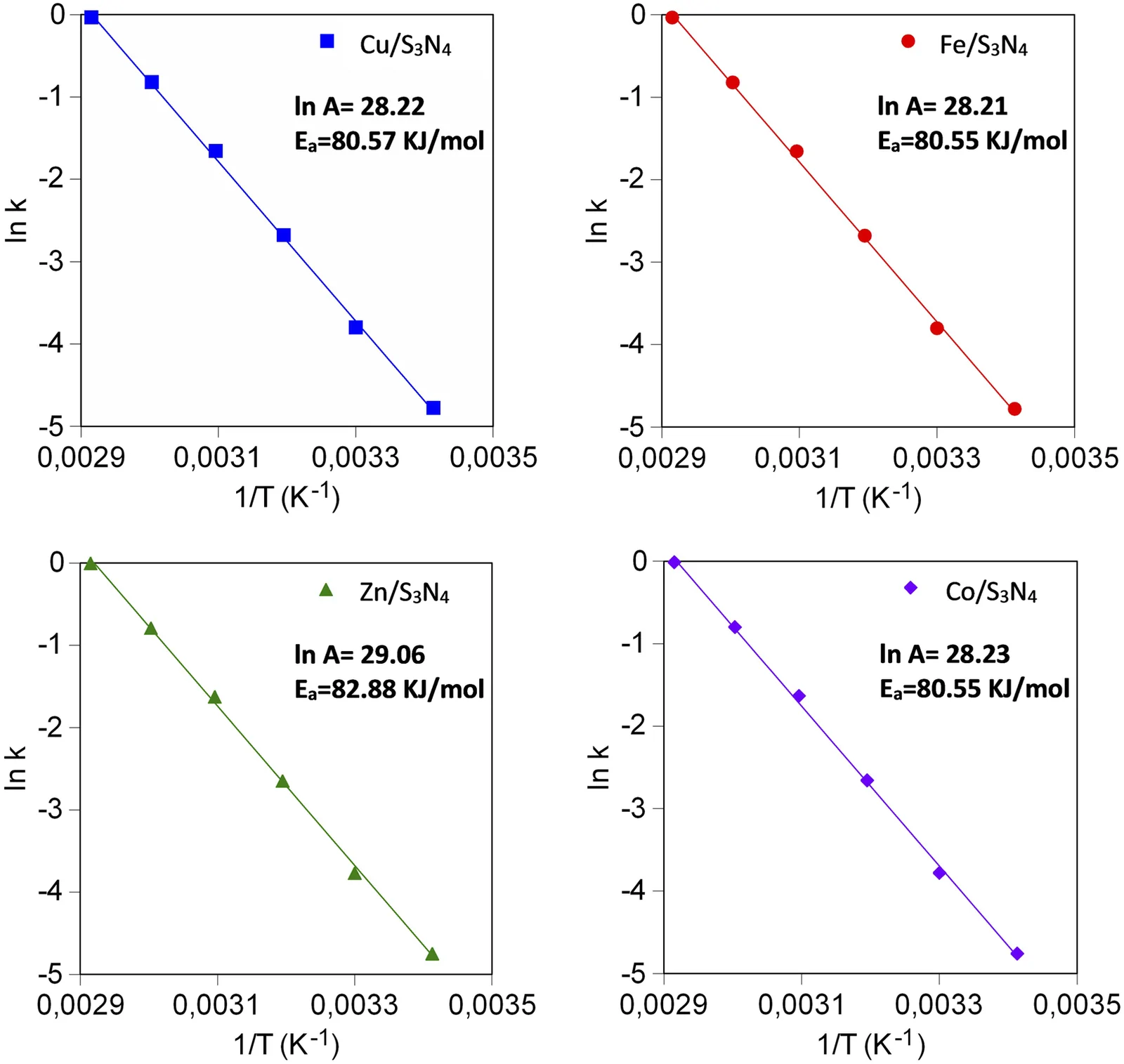
Arrhenius plot for MB degradation (reaction conditions: pH 6 for Cu/Si_3_N_4_ and pH 4 for Co/Si_3_N_4_, Fe/Si_3_N_4_ and Zn/Si_3_N_4_; catalyst dose 150 mg L^−1^ for Cu/Si_3_N_4_ and 300 mg L^−1^ for Co/Si_3_N_4_, Fe/Si_3_N_4_ and Zn/Si_3_N_4_; H_2_O_2_ dose 50 mM; MB 10 mg L^−1^; 70 °C; reaction time 30 minutes).

This indicates that despite different metals being loaded onto Si_3_N_4_, the kinetics are mainly governed by the support rather than the metal.^83^ The increase in the apparent rate constants with temperature is consistent with thermally accelerated reaction kinetics, resulting in enhanced MB degradation and mineralization at higher temperatures.^70,84^

### Catalyst recycling

Catalyst recyclability is a critical requirement for the practical implementation of heterogeneous Fenton systems, as highly active but poorly reusable catalysts are unsuitable for large-scale applications. In transition metal-based systems, catalytic stability is essential to prevent metal leaching and the associated risk of secondary pollution.^85,86^ Consequently, the long-term viability of heterogeneous Fenton catalysts depends on their ability to maintain catalytic activity over repeated cycles while limiting metal release into the treated solution. The recyclability of the M/Si_3_N_4_ catalysts was evaluated over five consecutive reaction cycles. After each run, the catalysts were recovered, washed with hot deionized water, dried at 120 °C, and reused under optimized reaction conditions. As shown in Fig. 11, the recycling performance strongly depends on the nature of the supported metal. The degradation kinetics of methylene blue (MB) across five cycles (R1–R5) reveal high retention of MB degradation activity for Cu/Si_3_N_4_, Fe/Si_3_N_4_, and Co/Si_3_N_4_.

**Fig. 11:**
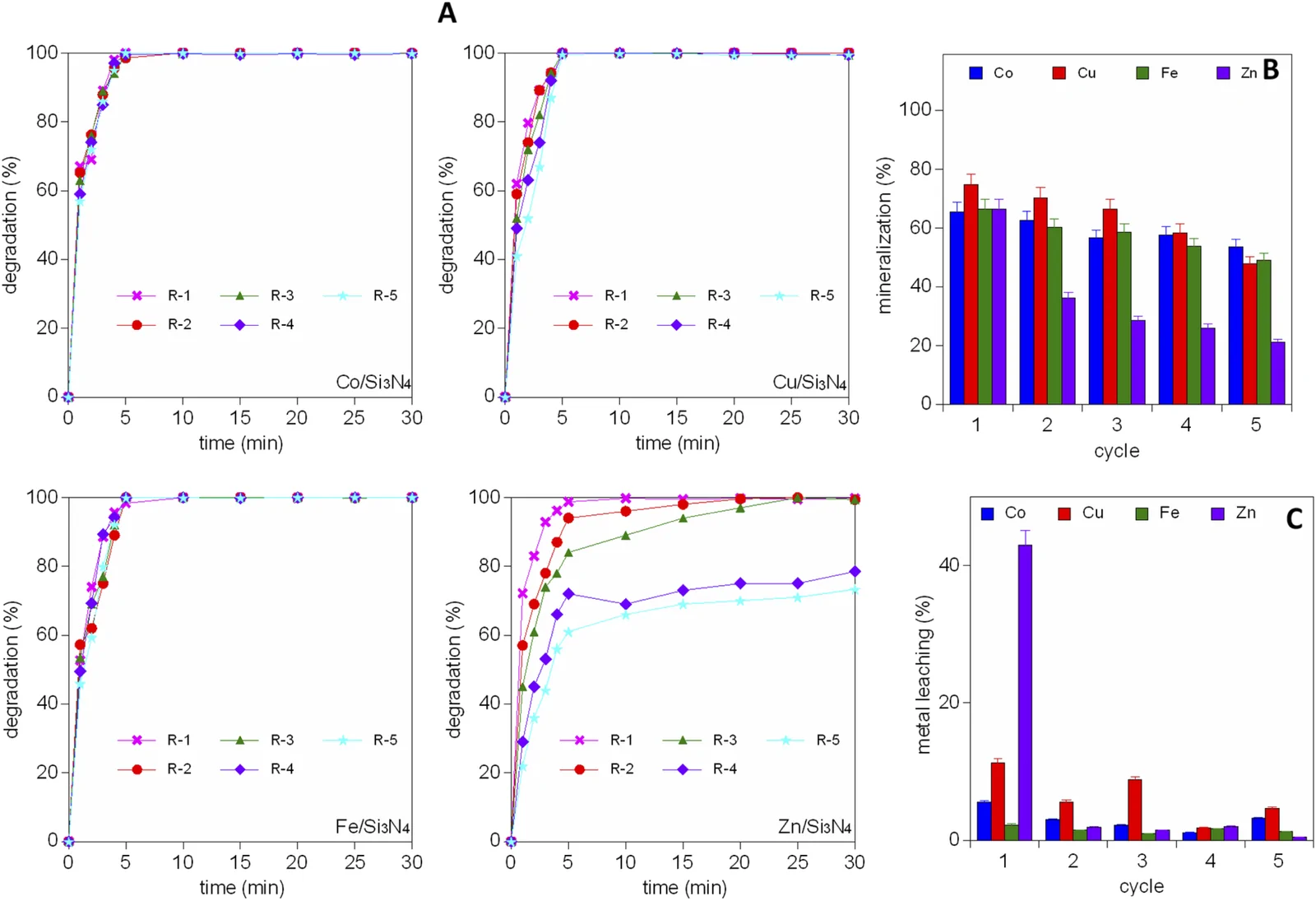
Effect of catalyst recycling on (A) MB degradation and (B) MB mineralization; (C) metal leaching after each cycle (reaction conditions: pH 6 for Cu/Si_3_N_4_ and pH 4 for Co/Si_3_N_4_, Fe/Si_3_N_4_ and Zn/Si_3_N_4_; catalyst dose 150 mg L^−1^ for Cu/Si_3_N_4_ and 300 mg L^−1^ for Co/Si_3_N_4_, Fe/Si_3_N_4_ and Zn/Si_3_N_4_; H_2_O_2_ dose 50 mM; MB 10 mg L^−1^; temperature 70 °C; reaction time 30 minutes).

For these catalysts, the degradation curves remain nearly superimposed throughout the recycling tests, consistently achieving complete MB removal within approximately 5 min. This behavior indicates that the active sites responsible for chromophore cleavage remain accessible and catalytically active during repeated use. In contrast, Zn/Si_3_N_4_ undergoes a pronounced loss of activity, with the degradation rate progressively decreasing and complete dye removal no longer achieved after 30 min in the fourth and fifth cycles. However, MB discoloration alone does not necessarily reflect the extent of mineralization of the organic matter. Total organic carbon (TOC) removal therefore provides complementary information on the ability of the catalysts to oxidize MB and its degradation intermediates. Cu/Si_3_N_4_ initially exhibits the highest mineralization efficiency (75%), which gradually decreases to approximately 48% after five cycles. Fe/Si_3_N_4_ and Co/Si_3_N_4_ display similar trends, maintaining mineralization efficiencies of 48–53% at the end of the recycling tests. Thus, although Cu/Si_3_N_4_, Fe/Si_3_N_4_, and Co/Si_3_N_4_ retain a high capacity for MB discoloration over five cycles, their mineralization efficiency decreases to some extent upon reuse. This difference between MB removal and TOC removal indicates that the catalysts retain their ability to cleave the chromophoric structure of MB, while their overall oxidation capacity toward organic intermediates is progressively reduced. Such behavior may be associated with partial surface fouling and/or accumulation of degradation by-products that can temporarily block or modify catalytically active sites. In contrast, Zn/Si_3_N_4_ shows a dramatic decrease in mineralization efficiency from 66% to only 21% by the fifth cycle, indicating a substantial loss of oxidative capacity toward intermediate organic species. Metal leaching analysis provides key insight into the observed differences in recycling performance (Fig. 11C and Table 4S). The poor recyclability of Zn/Si_3_N_4_ is primarily associated with severe metal leaching during the first cycle (43%), indicating weak anchoring of Zn species to the Si_3_N_4_ support. Once dissolved into the reaction medium, the number of heterogeneous active sites is irreversibly reduced, leading to rapid catalytic deterioration. Conversely, Cu/Si_3_N_4_, Fe/Si_3_N_4_, and Co/Si_3_N_4_ exhibit significantly greater structural stability. Although Cu/Si_3_N_4_ shows moderate initial leaching (11%), the metal release stabilizes in subsequent cycles, while Fe/Si_3_N_4_ and Co/Si_3_N_4_ maintain leaching levels consistently below 5%, ensuring that the process remains predominantly heterogeneous and environmentally acceptable. These results indicate that strong metal–nitride interactions contribute to stabilizing the active centers and preventing excessive dissolution under Fenton-like reaction conditions. Overall, the recycling study demonstrates that Si_3_N_4_ provides an effective support for stabilizing Cu, Fe, and Co active sites, enabling sustained catalytic activity with limited metal leaching. Among the investigated materials, Cu/Si_3_N_4_ emerges as the most efficient and robust catalyst, combining rapid degradation kinetics, high mineralization efficiency, and acceptable structural stability over repeated cycles. Conversely, the severe leaching and rapid performance decline of Zn/Si_3_N_4_ highlight the need for improved stabilization strategies before zinc-based nitride catalysts can be considered for practical environmental applications. These findings highlight that controlling metal-nitride interactions is a key factor in achieving high catalytic activity in heterogeneous Fenton systems. These insights provide valuable guidelines for the rational design of durable catalysts for advanced oxidation processes in wastewater treatment. Among the investigated materials, Cu/Si_3_N_4_ emerges as the most promising candidate for practical heterogeneous Fenton applications.

## Comparative assessment with literature

A rigorous evaluation of catalytic performance requires comparison with state-of-the-art heterogeneous Fenton catalysts reported in the literature. To better evaluate the catalytic performance of the M/Si_3_N_4_ materials, a comparative analysis with representative heterogeneous Fenton catalysts reported in recent literature was performed. The main operational parameters and catalytic efficiencies for MB degradation are summarized in Table 2.

**Table 2:** Comparison with literature results for MB removal over heterogeneous Fenton process

Catalyst	Catalyst (g L^−1^)	H_2_O_2_ (mM)	MB (mg L^−1^)	MB degradation		MB mineralization		Ref.
				(%)	Time (min)	(%)	Time (min)	
Cu/Si_3_N_4_	0.15	50	10	100	5	80	30	This work
Fe/Si_3_N_4_	0.30	50	10	100	5	65	30	This work
Co/Si_3_N_4_	0.30	50	10	100	5	62	30	This work
Zn/Si_3_N_4_	0.30	50	10	100	5	60	30	This work
MPCMSs	2	16	40	∼100	25	—	—	73
CuFe_2_O_4_/Cu@C	0.5	16	20	∼100	30	—	—	87
Fe_3_O_4_/C	0.5	90	10	∼100	60	—	—	88
Fe–Cu catalyst	0.5	25	5	∼100	60	—	—	89
Ferrocene	0.372	23.58	10	∼100	120	—	—	82
Fe_3_O_4_/usGO	0.0029	10	5	∼100	120	—	—	15
Fe_3_O_4_/rGO	0.3	60	10	98.6	120	—	—	86
N,C/CuO–Fe_2_O_3_	0.1	60	180	98	180	—	—	90
MnMg/Fe	2	13	20	96	300	—	—	91
Mg/Fe	2	13	20	80	300	—	—	91
Fe_3_O_4_/TiO_2_/C	1	100	50	80	140	—	—	92
FeNi/C-300	1	100	30	80	60	—	—	93
CuFe_2_O_4_	0.1	10	50	74	50	—	—	94

One of the most significant differences lies in the reaction kinetics. Several reported catalysts, including Fe_3_O_4_/rGO^86^ and N,C/CuO–Fe_2_O_3_,^90^ require relatively long reaction times (typically 120–180 min) to achieve high degradation efficiencies. In contrast, the M/Si_3_N_4_ catalysts investigated in this study achieve complete MB discoloration within approximately 5 min under optimized conditions. Even when compared with relatively fast systems such as MPCMSs (25 min),^73^ the present catalysts exhibit substantially faster reaction kinetics. This enhanced performance can be attributed to the nitrogen-rich environment of the Si_3_N_4_ support, which may promote efficient electron transfer and facilitate the activation of H_2_O_2_, thereby accelerating reactive oxygen species generation. Another important aspect concerns the evaluation of mineralization efficiency. Many studies focus primarily on dye discoloration, which reflects chromophore degradation but does not necessarily indicate complete oxidation of organic intermediates. As shown in Table 2, most of the reported systems do not provide total organic carbon (TOC) data, making it difficult to assess the extent of pollutant mineralization and the potential formation of intermediate by-products. In contrast, the catalysts developed in this work exhibit significant mineralization capacity, with Cu/Si_3_N_4_ achieving up to 80% TOC removal under optimized conditions. This result indicates not only efficient dye degradation but also substantial oxidation of intermediate organic species.

In addition to catalytic efficiency, operational parameters such as catalyst dosage are crucial for practical applications. The M/Si_3_N_4_ catalysts operate at relatively low dosages (0.15–0.30 g L^−1^), which are lower than those reported for several literature systems that require catalyst loadings up to 2.0 g L^−1^ to achieve comparable or lower degradation efficiencies.^91^ Among the investigated materials, Cu/Si_3_N_4_ shows the most favorable combination of rapid reaction kinetics, high mineralization efficiency, and low catalyst dosage. Overall, the comparative analysis suggests that Si_3_N_4_-supported transition metals represent a promising class of heterogeneous Fenton catalysts. By combining rapid degradation kinetics with significant mineralization capacity, these materials address some of the key limitations commonly reported for oxide-supported systems. In particular, the performance of Cu/Si_3_N_4_ highlights the potential of nitride-based supports for the development of efficient catalysts for advanced oxidation processes in wastewater treatment.

## Conclusions

In this work, Si_3_N_4_-supported transition metal catalysts (Cu, Fe, Co, Zn) were successfully synthesized and investigated for the first time in the heterogeneous Fenton-like oxidation of Methylene Blue. All M/Si_3_N_4_ formulations demonstrated ultra-fast degradation kinetics, achieving 100% discoloration within only 5 minutes. Unlike many conventional systems reported in the literature, these catalysts achieved high levels of mineralization (60–80%), ensuring a genuine reduction in effluent organic load. To quantitatively assess catalytic efficiency, a new descriptor termed the Heterogeneous Fenton Potential (HFP) was introduced. This parameter reflects the efficiency of oxidant-to-radical conversion and provides a useful tool for comparing catalytic systems and evaluating their potential feasibility. Among the investigated materials, Cu/Si_3_N_4_ exhibited the highest HFP value (1.71), indicating superior efficiency in H_2_O_2_ activation. Its ability to maintain peak performance at near-neutral pH (up to pH 6) represents a promising novelty, overcoming the “pH-acidic bottleneck” that typically limits industrial Fenton applications. This superior activity is intrinsically linked to the high dispersion of CuO nanoparticles (30–40 nm) and the unique electronic environment provided by the nitride support. Recyclability tests confirmed excellent structural stability and minimal metal leaching for Cu, Fe, and Co catalysts, whereas Zn/Si_3_N_4_ suffered rapid deactivation. These findings highlight the importance of metal–support compatibility. In conclusion, Si_3_N_4_-based materials represent a new class of sustainable and high-performance supports for advanced oxidation processes. The Cu/Si_3_N_4_ system, in particular, demonstrates promising potential for advanced oxidation processes, combining high catalytic activity, good recyclability, and limited metal release. These results support its further investigation for the treatment of real industrial wastewaters, where long-term stability, mineralization performance, and process-scale energy requirements should be further assessed.

## Author contributions

Sajid Hussain: investigation, data curation, writing—original draft preparation. Eleonora Aneggi: conceptualization, methodology, writing—original draft preparation, writing—review and editing. Walter Baratta: writing—review and editing. Daniele Zuccaccia: writing—review and editing. Jordi Llorca: investigation, writing—review and editing. Alessandro Trovarelli: writing—review and editing. Michele Modesti: writing—review and editing. Daniele Goi: writing—review and editing.

## Conflicts of interest

There are no conflicts to declare.

## Supplementary Material

RA-OLF-D6RA06509K-s001

## Data Availability

The data supporting the findings of this study are available within the article and its supplementary information (SI). Supplementary information is available. See DOI: https://doi.org/10.1039/d6ra06509k.
