# Supplementary material for: pH-resilient Cu/Si3N4 catalyst for efficient heterogeneous Fenton mineralization of methylene blue
Source: RSC Adv. 2026 Oct 23. Online ahead of print. doi: 10.1039/d6ra06509k (PMC13692716; doi:10.1039/d6ra06509k)
Supplement: Supplementary file 1 [file RA-OLF-D6RA06509K-s001.pdf]

Supplementary Information (SI) for RSC Advances.
This journal is © The Royal Society of Chemistry

Supplementary Information

pH-Resilient Cu/Si_3N_4 Catalyst for Efficient Heterogeneous Fenton Mineralization of Methylene Blue

Sajid Hussain[1,1], Eleonora Aneggi[2,*], Walter Baratta[2], Daniele Zuccaccia[2], Jordi Llorca[3] Alessandro Trovarelli[1], Michele Modesti[4], Daniele Goi[1]

[1] Dipartimento Politecnico di Ingegneria e Architettura, Università di Udine, e INSTM Udine, via del Cotonificio 108, 33100, Udine, Italy (hussain.sajid@spes.uniud.it, alessandro.trovarelli@uniud.it, daniele.goi@uniud.it);

[2] Dipartimento di Scienze AgroAlimentari, Ambientali e Animali, Sezione di Chimica, Università di Udine e INSTM Udine, via del Cotonificio 108, 33100, Udine, Italy (walter.baratta@uniud.it, daniele.zuccaccia@uniud.it);

[3] Department of Chemical Engineering and Barcelona Research Center in Multiscale Science and Engineering, Universitat Politècnica de Catalunya, EEBE, Eduard Maristany 10-14, 08019 Barcelona, Spain (jordi.llorca@upc.edu)

[4] Dipartimento di Ingegneria Industriale, Università di Padova, Via Gradenigo, 6/a - 35131 Padova, Italy (michele.modesti@unipd.it)

[*] Correspondence: eleonora.aneggi@uniud.it (E.A.)

[1] Present Address: Sajid Hussain, Dipartimento di Ingegneria Industriale, Università di Padova, via Marzolo 9, 35131, Padova, Italy

Results and discussion

Catalyst Characterization

Table 1S. Assignment of main signals in the Raman spectra of the investigate samples.

Raman shift (cm^{-1})	assignment
182	b-Si_3N_4
206	b -Si_3N_4
225	b -Si_3N_4
230	Fe_2O_3
248	Fe_2O_3
290	CuO
293	Fe_2O_3
412	Fe_2O_3
450	b -Si_3N_4
470	Co_3O_4
515	Co_3O_4
517	Si_2N_2O
615	b -Si_3N_4
675	Co_3O_4
730	b -Si_3N_4
860	b -Si_3N_4
928	b -Si_3N_4
940	b -Si_3N_4
1045	v -Si_3N_4

Table 2S. Blank and control experiments

Trial	Degradation (%)	Mineralization(%)
MB + M/Si_3N_4	5-7	2-4
MB + H_2O_2	13	7
MB + Bare Si_3N_4 + H_2O_2	34	9

Table 3S. Values used for the calculation of the Heterogeneous Fenton Potential (HFP)

Sample	O_f/O_i	C_f/C_i	$1 - O_f/O_i$	G	HPF
Ideal	1	1	0	0	2,00
Co/Si_3N_4	0,9667	0,751	0,033	0,2157	1,71
Cu/Si_3N_4	0,8667	0,664	0,133	0,2027	1,50
Fe/Si_3N_4	0,9	0,663	0,1	0,237	1,54
Zn/Si_3N_4	0,7667	0,654	0,233	0,1127	1,39

Reaction kinetics of MB degradation

Equations 3S-5S for conventional kinetic models.

$$\frac{dC}{dt} = -k_0 \quad \text{Eq. 1S}$$

$$\frac{dC}{dt} = -k_1[C] \quad \text{Eq. 2S}$$

$$\frac{dC}{dt} = -k_2[C]^2 \quad \text{Eq. 3S}$$

where:

C_0 = Initial MB concentration

C_t = MB concentration after specific time interval

k_0 = Zero-order rate constant

k_1 = First-order rate constant

k_2 = Second-order rate constant

The integration of Eq. 1S-3S give rise to the fundamental integrated laws, represented by Eq. 4S-6S, which we used for the determination of the order of the reaction and rate of reaction.

$$C_t - C_0 = -k_0 t \quad \text{Eq. 4S}$$

$$\ln\frac{C_t}{C_0} = -k_1 t \quad \text{Eq. 5S}$$

$$\frac{1}{C_t} - \frac{1}{C_0} = +k_2 t \quad \text{Eq. 6S}$$

The Arrhenius equation is reported in Eq. 7S.

$$k = A \exp[\frac{-E_a}{RT}] \qquad \text{Eq. 7S}$$

where:

k = Kinetic reaction constant

A = Preexponential factor (Arrhenius constant)

E_a = Activation energy

R = ideal gas constant

T = absolute temperature

The integrated form of Eq. 7S gives Eq. 8S:

$$\ln k = \ln A - \frac{E_a}{RT} \qquad \text{Eq. 8S}$$

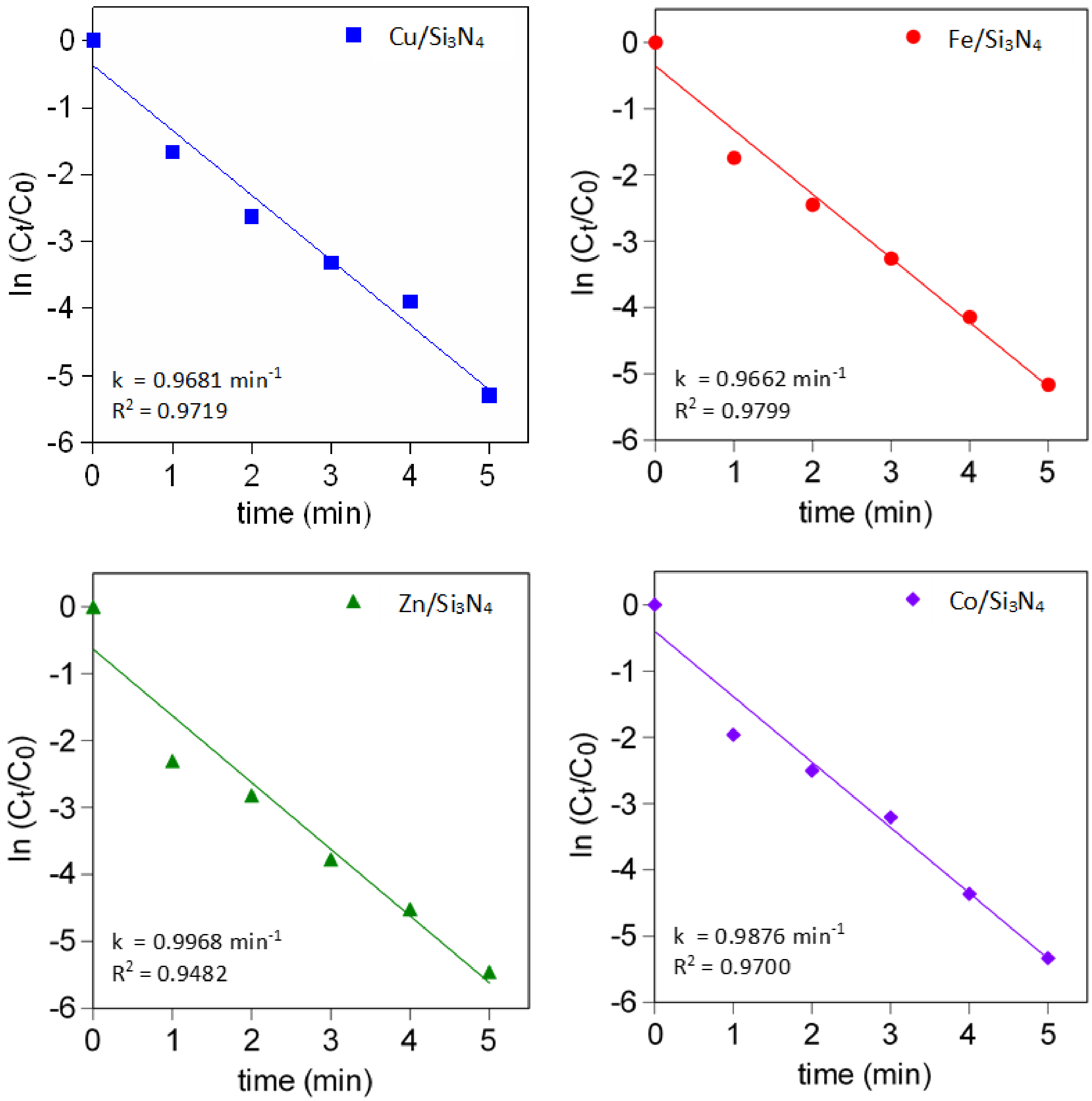

Figure 1S. MB degradation kinetics for all the employed catalysts (reaction conditions: pH 6 for Cu/Si_3N_4 and pH 4 for Co/Si_3N_4, Fe/Si_3N_4 and Zn/Si_3N_4; catalyst dose 150 mg/L for Cu/Si_3N_4 and 300 mg/L for Co/Si_3N_4, Fe/Si_3N_4 and Zn/Si_3N_4; H_2O_2 dose 50 mM; MB 10 mg/L; temperature 70 °C; reaction time 0-5 minutes).

Table 4S. First-cycle metal leaching and corresponding dissolved metal concentrations

Catalyst	Metal	Leaching (%)	Dissolved metal (mg/L)
Co/Si_3N_4	Co	5.53	0.83
Cu/Si_3N_4	Cu	11.29	0.85
Fe/Si_3N_4	Fe	2.27	0.34
Zn/Si_3N_4	Zn	42.93	6.44

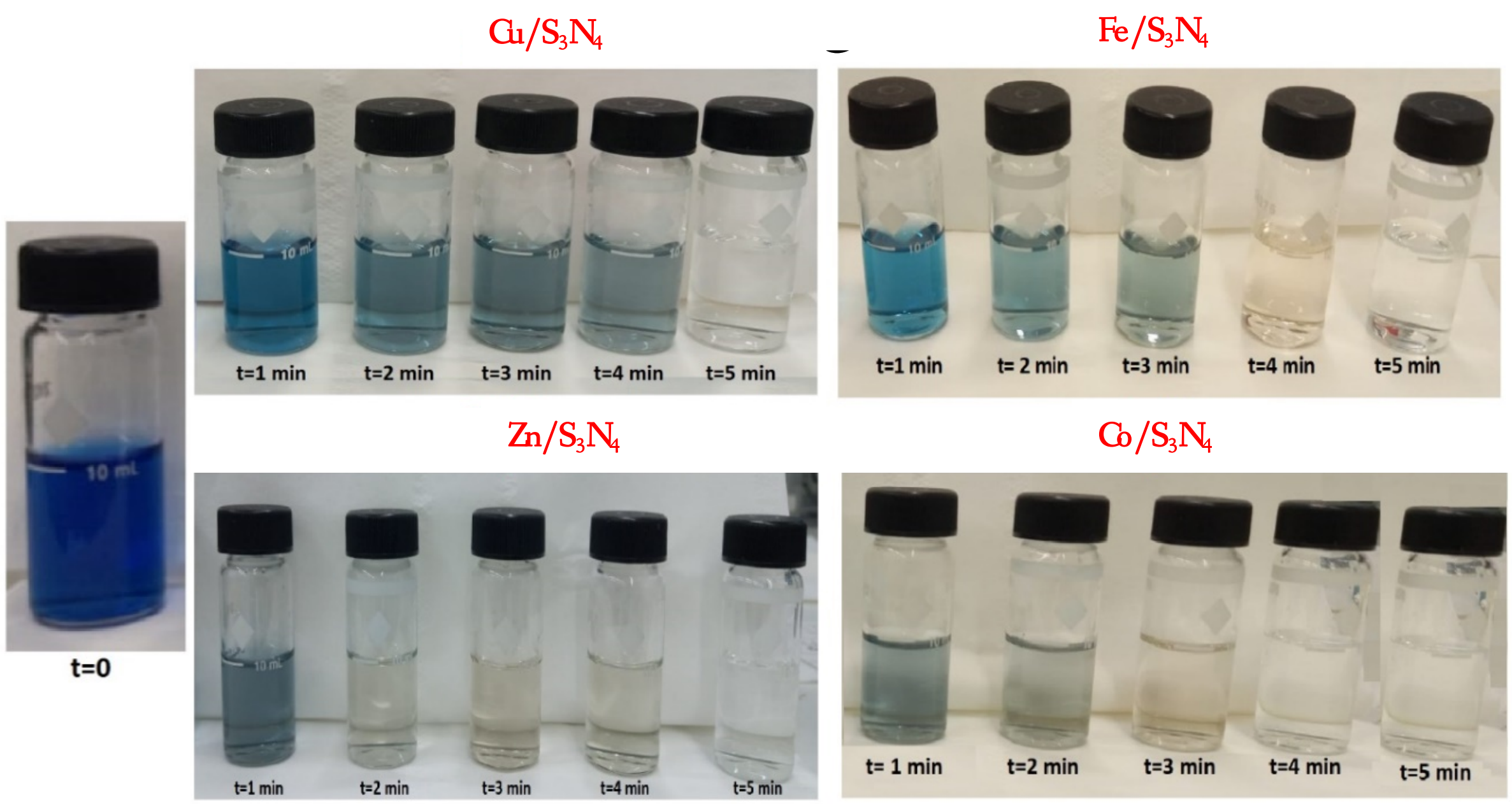

Figure 2S. Photographic evidence of methylene blue (MB) degradation during heterogeneous Fenton oxidation process (reaction conditions: pH 6 for Cu/Si_3N_4 and pH 4 for Co/Si_3N_4, Fe/Si_3N_4 and Zn/Si_3N_4; catalyst dose 150 mg/L for Cu/Si_3N_4 and 300 mg/L for Co/Si_3N_4, Fe/Si_3N_4 and Zn/Si_3N_4 H_2O_2 dose 50 mM; MB 10 mg/L; temperature 70 °C; reaction time 5 minutes).
